# Pathomechanisms of ALS8: altered autophagy and defective RNA binding protein (RBP) homeostasis due to the VAPB P56S mutation

**DOI:** 10.1038/s41419-021-03710-y

**Published:** 2021-05-10

**Authors:** Priyanka Tripathi, Haihong Guo, Alice Dreser, Alfred Yamoah, Antonio Sechi, Christopher Marvin Jesse, Istvan Katona, Panagiotis Doukas, Stefan Nikolin, Sabrina Ernst, Eleonora Aronica, Hannes Glaß, Andreas Hermann, Harry Steinbusch, Alfred C. Feller, Markus Bergmann, Dick Jaarsma, Joachim Weis, Anand Goswami

**Affiliations:** 1 Institute of Neuropathology, RWTH Aachen University Medical School, Pauwelsstr. 30, 52074 Aachen, Germany; 2 EURON - European Graduate School of Neuroscience, Maastricht, The Netherlands; 3 Institute of Biomedical Engineering, Department of Cell Biology, RWTH Aachen University Medical School, Pauwelsstr. 30, 52074 Aachen, Germany; 4 Department of Neurosurgery, Inselspital, Bern University Hospital, University of Bern, Bern, Switzerland; 5 Institute of Biochemistry and Molecular Biology, RWTH Aachen University, 52074 Aachen, Germany; 6 Confocal Microscopy Facility, Interdisciplinary Center for Clinical Research IZKF, RWTH Aachen University, 52074 Aachen, Germany; 7 Amsterdam UMC, University of Amsterdam, Department of (Neuro) Pathology, Amsterdam Neuroscience, Meibergdreef 9, 1105 AZ Amsterdam, The Netherlands; 8 Translational Neurodegeneration Section “Albrecht-Kossel”, Department of Neurology and Center for Transdisciplinary Neurosciences Rostock (CTNR), University Medical Center Rostock, University of Rostock, 18147 Rostock, Germany; 9 German Center for Neurodegenerative Diseases (DZNE) Rostock/Greifswald, 18147 Rostock, Germany; 10 Department of Psychiatry and Neuropsychology, School for Mental Health and Neuroscience, Maastricht University, Maastricht, The Netherlands; 11 Hämatopathologie Lübeck, Reference Centre for Lymph Node Pathology and Haematopathology, University Hospital of Schleswig-Holstein, Lübeck, Germany; 12 Institute für Neuropathologie, Bremen-Mitte, Bremen, Germany; 13 Department of Neuroscience, Erasmus MC, Rotterdam, 3015 GD The Netherlands

**Keywords:** Mechanisms of disease, Amyotrophic lateral sclerosis

## Abstract

Mutations in RNA binding proteins (RBPs) and in genes regulating autophagy are frequent causes of familial amyotrophic lateral sclerosis (fALS). The P56S mutation in vesicle-associated membrane protein-associated protein B (VAPB) leads to fALS (ALS8) and spinal muscular atrophy (SMA). While VAPB is primarily involved in the unfolded protein response (UPR), vesicular trafficking and in initial steps of the autophagy pathway, the effect of mutant P56S-VAPB on autophagy regulation in connection with RBP homeostasis has not been explored yet. Examining the muscle biopsy of our index ALS8 patient of European origin revealed globular accumulations of VAPB aggregates co-localised with autophagy markers LC3 and p62 in partially atrophic and atrophic muscle fibres. In line with this skin fibroblasts obtained from the same patient showed accumulation of P56S-VAPB aggregates together with LC3 and p62. Detailed investigations of autophagic flux in cell culture models revealed that P56S-VAPB alters both initial and late steps of the autophagy pathway. Accordingly, electron microscopy complemented with live cell imaging highlighted the impaired fusion of accumulated autophagosomes with lysosomes in cells expressing P56S-VAPB. Consistent with these observations, neuropathological studies of brain and spinal cord of P56S-VAPB transgenic mice revealed signs of neurodegeneration associated with altered protein quality control and defective autophagy. Autophagy and RBP homeostasis are interdependent, as demonstrated by the cytoplasmic mis-localisation of several RBPs including pTDP-43, FUS, Matrin 3 which often sequestered with P56S-VAPB aggregates both in cell culture and in the muscle biopsy of the ALS8 patient. Further confirming the notion that aggregation of the RBPs proceeds through the stress granule (SG) pathway, we found persistent G3BP- and TIAR1-positive SGs in P56S-VAPB expressing cells as well as in the ALS8 patient muscle biopsy. We conclude that P56S-VAPB-ALS8 involves a cohesive pathomechanism of aberrant RBP homeostasis together with dysfunctional autophagy.

## Introduction

Amyotrophic lateral sclerosis (ALS) is a devastating disease characterised by progressive loss of upper and lower motor neurons (MNs), eventually leading to paralysis and death. Majority of the fALS- linked genes either regulate protein quality control and autophagy and/or RNA binding protein (RBP) homeostasis^1,2^. Pathogenic mutations in these genes induce neuronal toxicity coupled with protein aggregation, defective proteostasis/autophagy and aberrant RBP homeostasis. However, the factors that initially triggers these key events and the functional interplay between autophagy and RBPs homeostasis and their downstream signalling are poorly understood thus far.

A dominantly inherited mutation (P56S) in the gene encoding for the vesicle-associated membrane protein-associated protein B (VAPB) has been associated with typical ALS (ALS8), atypical ALS and late-onset spinal muscular atrophy (SMA)^3–5^. VAPB is a ubiquitously expressed membrane-anchored protein of ER and ER-Golgi intermediate vesicles^6,7^. VAPB contains an N-terminal major sperm protein (MSP), a coiled coil and a transmembrane domain. The MSP domain binds to a variety of proteins from various cellular compartments that contain FFAT motifs (two phenylalanines in an acidic region) and that are known to orchestrate multiple functions including retrograde transport of proteins^6^, lipid transfer toward the Golgi^8^, regulation of ER structure through interaction with the microtubule network^9,10^ and modulation of responses to ER stress^11–13^. In line with this, recent studies have shown that VAPB is involved in ER-orchestrated autophagosome biogenesis^14^.

P56S-VAPB forms detergent insoluble cytoplasmic inclusions, which exhibits toxic gain of functions as shown by others^12,13,15–18^ including the detrimental effects of aggregated P56S-VAPB on protein quality control, ER-organelle tethering, mitochondrial toxicity, UPR imbalance and alterations in endo-lysosomal pathways^11,13,19–21^. P56S-VAPB transgenic (Tg) mice under the control of a pan-neuronal promoter Thy1.2 showed signs of ER stress in both spinal MNs as well as in CNS neurons accompanied by a minor degeneration of CNS neurons^22^. Lumbar spinal cord α-MNs of these mice can develop cytoplasmic TDP-43 accumulation^23^, but showed only incomplete degeneration accompanied by a mild motor phenotype at 18 months of age^23^. In line with this observation, P56S-VAPB knock-in mice displayed induction of ER stress and autophagic responses in α-MNs before obvious onset of behavioural defects^24^. In addition to the toxic gain of function P56S-VAPB has been shown to induce a dominant negative mechanism by incorporating the normal wild-type (wt) VAPB, supporting a loss of function hypothesis for ALS8 pathogenesis^13,16,17^. Consistent with these data, VAPB knockdown in zebrafish led to swimming deficits and VAPB knockout mice showed mild motor deficits associated with autophagy defects^25^. Taken together, these results suggest that VAPB is involved in many functions which are crucial for neuronal survival and that P56S-VAPB alters these processes involving both toxic gain and/or loss of function. However, it is still not clear how these pathways are interconnected and pathologically orchestrated in ALS8 to induce neurodegeneration and if and how P56S-VAPB modulates these converging mechanisms.

**Scheme 1:**
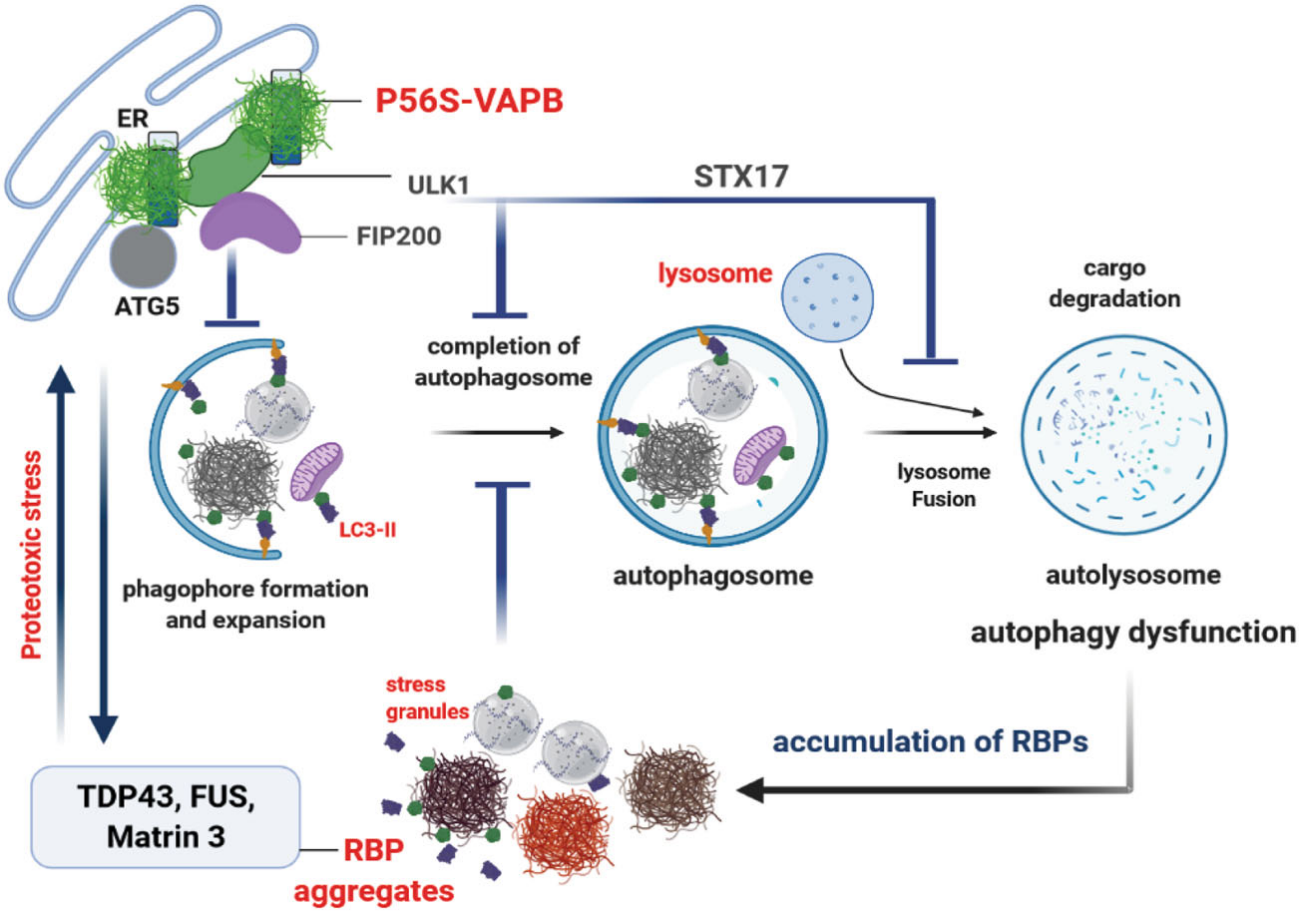
Proposed cohesive pathomechanism of aberrant RBPs hoemostasis and autophagy in ALS8. P56S-VAPB aggregates alter early autophagy proteins ULK1, ATG5-ATG12; late autophagy protein STX17 and leads to the defects in both early and late steps of autophagy. P56S-VAPB mediated proteotoxic stress together with altered autophagy might contribute to dysregulations of several fALS-associated RBPs (TDP-43, FUS, Matrin 3) including the SGs and can initiate a vicious cycle. (Schematic image was generated with the BioRender software).

To address these issues, we focussed on the effect of P56S-VAPB on autophagy and RBP homeostasis. We found that P56S-VAPB-mediated ALS pathology is closely linked to alterations of both of these interdependent cellular processes. Motor neurons are known to be particularly vulnerable to altered autophagy and RBP homeostasis^1,26–30^, explaining why they are selectively affected by a mutation that is altering these pathways simultaneously (see also schematic representation, Scheme 1)

## Materials and methods

### Reagents and antibodies

Fluorescent nucleic acid stain Hoechst 33258 was purchased from Molecular Probes. Thapsigargin, MG132, Tunicamycin, EGF, Rapamycin, Bafilomycin A, HEPES 4-(2-hydroxyethyl)-1-piperazineethanesulfonic acid were purchased from Sigma Aldrich. All primary and secondary antibodies and their dilutions used in this study are listed in Supplementary Table 1. Many of these antibodies have been used by us in previous published studies (see references in Supplementary Table 1). Rabbit polyclonal VAPB antibody was custom made and used by us in previous studies^19,31–33^

### Human muscle biopsies

Autopsy tissue from P56S-VAPB is not available so far. We could use archival, formalin-fixed, paraffin-embedded muscle biopsy materials obtained from an index patient from European origin with ALS8 due to the P56S-VAPB mutation^5^ and stain cryostat sections and resin blocks of glutaraldehyde-fixed muscle tissue from his mother, who had shown similar symptoms as her son, but had died before genetic diagnosis had been available. Muscle biopsies from clinically confirmed sporadic ALS as well as age-matched control muscle biopsies were also obtained from the archives of the Institute of Neuropathology, RWTH Aachen University Hospital, following the guidelines of the Ethics Committee of RWTH Aachen University Hospital (also see below ethical statement).

### Experimental animals (P56S-VAPB transgenic mice)

VAPB transgenic mice had been generated using the cDNAs of wild-type or P56S-mutant human *VAPB* cloned into the Thy1.2-expression module. The VAPB constructs also contained an HA-tag to enable easy visualisation of transgenic VAPB by immunocytochemical and biochemical approaches. Details of the generation of this transgenic mouse are described elsewhere^15^. In this study we used tissue from Thy1 wt-VAPB-HA and Thy1 P56S-VAPB-HA mice at the age around 200 days. Animals were not randomly assigned during collection, but the strain, sex, and age of the mice were the same. We used paraffin sections of formalin-fixed material from these animals for most of the histological analyses and fresh frozen tissue for Western blot analyses. Immunohistochemistry protocols for formalin-fixed, paraffin-embedded mouse tissue and all the antibodies used (Supplementary Table 1) were optimised and performed in analogy to the protocol used for human biopsy and autopsy tissue to avoid any discrepancies. All procedures including housing and handling the animals were done in accordance with the “Principles of laboratory animal care” (NIH publication No. 86–23) and the guidelines of the Erasmus University (The Netherlands) animal care committee.

### Cell culture, transient transfection and treatments

#### Cell culture and treatment

Human epithelial cancer cells (HeLa), Human embryonic kidney cell line (HEK-293), African green monkey kidney cells (COS-7) and NSC34 motor neuron-like cells were cultured in Dulbecco’s modified Eagle’s medium (DMEM, Invitrogen, Carlsbad, CA, USA), supplemented with 10% FBS and 1% antibiotic/anti-mycotic solution (Invitrogen). Mouse embryonic fibroblasts (MEF) obtained from the GFP-LC3 transgenic mice^34^ were also maintained in DMEM supplemented with 10% FBS and 1% antibiotic/anti-mycotic solution. The human epidermoid carcinoma cell line A431 was grown in Dulbecco’s modified Eagle’s medium (DMEM, Invitrogen, Carlsbad, CA, USA) supplemented with 10% FBS and 0.1% Gentamycin. NIH-3T3 cells stably expressing GFP-LC3 or tandem mCherry-EGFP-LC3 were cultured in DMEM supplemented with 10% FBS, 1% penicillin/streptomycin and puromycin (Sigma Aldrich). Fibroblasts obtained from P56S-ALS8 fALS patients and healthy controls were cultured in Dulbecco’s modified Eagle’s medium (DMEM, Invitrogen, Carlsbad, CA, USA), supplemented with 10% FBS and 1% antibiotic/anti-mycotic solution (Invitrogen) 20% FBS, 1% penicillin/streptomycin, 1% l-Glutamin and 0.056% amphotericin. Cells were maintained in a humidified incubator at 37 °C and 5% CO_2_. Generation of NIH-3T3 cells stably expressing GFP-LC3 or tandem mCherry-EGFP-LC3 with retroviral infection is described elsewhere^35^.

### Human IPSc-derived motor neurons (MNs)

We used iPSCs generated from fibroblasts of a healthy individual donor (male, age at skin biopsy 34). The generation and characterisation of the line was previously described^36^. For MNs differentiation, we first derived neural precursor cells (NPCs) which were maintained and differentiation into MNs as shown previously^37^. In brief, NPC were maintained in basic medium (DMEM-F12/Neurobasal 50:50 medium, N2 Supplement (1:200), B27 Supplement without vitamin A (1:100), penicillin/streptomycin (1%), GlutaMAX (1%)), supplemented with Chiron 99021 (3 μM), Ascorbic acid (150 μM) and Purmorphamine (0.5 μM) on the dishes coated with Matrigel. For induction of the differentiation into motor neurons, NPC were split on the Matrigel coated dish in the basic medium supplemented with BDNF (1 ng/ml), Ascorbic acid (200 μM), Retinoic acid (1 μM), GDNF (1 ng/ml) and Purmorphamine (0.5 μM) and maintained for 5 days. For the final maturation, the medium was changed on the day 6 to the basic medium supplemented with DBcAMP (100 μM), BDNF (2 ng/ml), Ascorbic acid (200 μM), TGFβ-3 (1 ng/ml) and GDNF (2 ng/ml). Between day 7 and 10 the cells were split onto the dishes coated with Poly-L-ornithine and laminin and maintained for at least 4–5 weeks before they were used for the final analysis. The cells were regularly tested for mycoplasma contamination.

### Transient transfections

Cells were transfected to express either GFP-wt-VAPB or GFP-P56S-VAPB. The detailed description of generation of these plasmids is given elsewhere^31^. The empty pEGFP-N1 vector was used as a transfection control. All cell lines were transfected using Lipofectamine 2000 reagent (Invitrogen) according to the manufacturer’s recommendations. After 4 h incubation at 37 °C and 5% CO_2_ the transfection reagent containing medium was replaced by fresh medium and analysis was performed 48 h later. IPS-derived MNs were transfected using NeuroMag reagent (OZ Biosciences) according to the manufacturer’s protocol; the analysis was performed after 48 h.

### Immunocytochemistry

HeLa, HEK-293, COS-7, MEFs and NIH-3T3 cells were cultured on µ-dishes (ibidi, GmbH) and transiently transfected either with GFP-wt-VAPB or P56S-VAPB. After 48 h cells were fixed in 4% PFA and processed for confocal microscopy. Permeabilization with 0.5% Triton X100 and blocking with 4% skimmed milk/normal goat serum was followed by primary antibody incubation overnight at 4 °C. Secondary Alexa488- or Alexa594-conjugated anti-mouse or anti-rabbit antibodies (Invitrogen) were used for visualisation. Nuclei were stained with Hoechst 33258 (1 µg/ml) or were mounted with DAPI containing fluorescent mounting media (DAKO) and visualised using a Zeiss LSM 700 confocal microscope (Zeiss, Oberkochen, Germany). Images were processed using the Zeiss LSM software and Adobe Photoshop CS5.

### Stress granule analysis

FRAP experiments for stress granules analysis on RFP-G3BP cell lines^38,39^ overexpressing wt and P56S-VAPB were performed on a Zeiss LSM710 confocal microscope (Zeiss, Oberkochen, Germany) equipped with a cell incubator at 37 °C and 5% CO_2_. The images were acquired using a Plan-Apochromat ×63/1.40 Oil DIC M27 objective and analysed with ZEN 2012 software. For bleaching 90% power of the 561 nm laser (max. 20 mW) was used with the following capture settings: 380 ms per frame and one image every 600 ms with 10 and 100 frames before and after bleaching. The bleaching area covered 20 × 20 pixels with a pixel size of 0.1 µm. For all experiments the bleached area and the duration of the laser impulse were kept constant. The extent of recovery of the fluorescent signal was determined using ImageJ to measure the average pixel intensity values within three distinct regions of interest: ROI1: bleached area, ROI2: unbleached area within the cell and ROI3: background. Normalised FRAP recovery curves and the mobile fraction were calculated using the programme easy FRAP^40^.

### Live cell imaging to analyse RFP-GFP-LC3 fusion

To analyse the dynamics of the RFP-GFP-LC3 fusion protein, GFP and RFP channels were acquired every minute for up to 4 h using the imaging system described above, see also^35^. The extent of autophagosome maturation was determined by measuring the co-localisation of the GFP and RFP signals as expressed by the Pearson’s coefficient using ZEN software.

### Immunoblot analysis

Cells were washed twice with ice-cold PBS and scraped off the culture plate. After centrifugation at 6000 rpm for 5 min and removal of the supernatant, cell pellets were re-suspended in lysis buffer (50 mM Tris-Cl, pH8.0, 150 mM NaCl, 1% Triton X-100 in PBS, 0.5 mM PMSF and complete protease inhibitor mixture, Roche Applied Sciences) and incubated on ice for 30 min followed by sonication with an amplitude of 8% for 10 seconds. Clear lysates were obtained after centrifugation for 5 min at 6000 rpm. Protein concentrations were determined using the BCA method (Molecular Probes). Equal amounts of protein were boiled for 5 min in 2x SDS sample buffer and subjected to 10 or 12% SDS–PAGE electrophoresis at 20 mA/gel before being transferred to a polyvinylidenedifluoride (PVDF) membrane, which had to be activated in methanol before. Transfer lasted 1 h and 30 min at 350 mA and was followed by blocking in 4% skimmed milk in 0.08% Tween 20/Tris-buffered saline (TBS-T) for 30 min prior to incubation with primary antibody. The dilutions for primary antibodies are described in Supplementary Table 1. After incubating the primary antibody overnight at 4 °C under gently shaking, membranes were washed three times with TBS-T for 10 min each and incubated with the appropriate horseradish peroxidase-conjugated secondary antibody for 1 h (antibody dilution 1:10,000) followed by the same washing procedure. Immunoreactive proteins were detected by enhanced chemiluminescence (Amersham Biosciences). Densitometric quantification of the band intensity was normalised to tubulin levels using Adobe Photoshop CS5.

### EGFR endocytosis, starvation and degradation assay

For EGFR endocytosis and degradation analysis, A431 cells were starved in DMEM without serum for 4 h. After starvation, cells were treated with EGF (100 mg/ml) to stimulate EGFR endocytosis. Cells were then collected at various time points and lysed in RIPA buffer (50 mM Tris–HCl pH 7.5, 1% Triton X-100, 150 mM NaCl, 1 mM EDTA and 0.1% Na deoxycholate) containing protease inhibitor. Protein extracts were resolved by SDS–PAGE and immunoblotted using anti-EGFR antibody.

### EGFR surface biotinylation assay

Surface biotinylation assays were performed as previously described^35^ with minor modifications. Briefly, transfected A431 cells were starved in DMEM without serum for 4 h. After starvation, cells were treated with EGF to stimulate EGFR endocytosis and then preceded for surface biotinylation assays according to the manufacturer’s protocol.

### Subcellular fractionation

The subcellular distributions of distinct proteins were determined by using a subcellular fractionation kit following the manufacturer’s protocol (Thermo Scientific/Life Technologies) and analysed by immunoblotting. The subcellular fractionation was performed on HeLa cells as well as on NSC34 cells, both transfected with GFP-wt-VAPB or P56S-VAPB.

### Immunohistochemistry

#### Diaminobenzidine

In all, 3–4-µm paraffin sections were placed on poly-L-lysine coated slides and allowed to dry in an oven (37 °C) overnight and then processed for immunohistochemistry described in detail elsewhere^32^. Sections deparaffinized in xylene for 20 min were rehydrated in 100%, 95% and 70% ethanol for 5 min each followed by endogenous peroxidase quenching (0.3% H_2_O_2_ in methanol) for 20 min. For antigen retrieval, sections were heated in citrate buffer, pH 6 (DAKO), for 20 min in a pressure cooker. After washing in PBS, sections were incubated with primary antibody (Supplementary Table 1) for 1 h at room temperature or at 4 °C overnight. After washing in PBS, sections were incubated with appropriate polymeric HRP-linker secondary antibody (IL Immunologic, Duiven, The Netherlands) for 30 min at room temperature. Diaminobenzidine (DAB) reagent (DCS Innovative Diagnostic System DAB kit) was used to stain the sections which were then counter-stained with 6% hematoxylin for 3 min. All procedures were performed at room temperature. Standard histological and histochemical stains including H&E, modified Gomori trichrome, non-specific esterase and NADH were performed as described by us and others previously^41^.

#### Immunofluorescence

Single and double immunofluorescence staining was performed as described by us already elsewhere^32,42^. In brief, deparaffinized tissue sections were heated in citrate buffer, pH 6 (Dako), for 20 min in a pressure cooker. Sections were then blocked (to avoid non-specific bindings) with ready to use 10% normal goat serum (Life Technologies, MD, USA) for 1 h at room temperature before incubating with primary antibody at 4 °C overnight. After washing in TBS-T for 10 min, the sections were incubated with Alexa conjugated secondary antibody (1 : 500 in TBS-T) at room temperature for 2 h. Sections were washed in TBS-T (2 × 10 min) and stained for 10 min with 0.1% Sudan Black in 80% ethanol to suppress endogenous lipofuscin auto-fluorescence. Finally, the sections were washed for 5 min in TBST and mounted with Vectashield mounting medium (Vector Laboratories) containing DAPI.

#### Transmission electron microscopy (TEM)

NSC-34 cells were transfected with pEGFP-N1, GFP-wt-VAPB or P56S-VAPB as described above. Cells were collected by scraping and then washed in 0.1 M phosphate buffer and immediately fixed with 2.5% glutaraldehyde in 0.1 M phosphate buffer for 24 h followed by washing in buffer for another 24 h. Cell pellets were collected by centrifugation (1000 rpm, 5 min) and embedded in 2% agarose (at 60 °C; Fluka # 05073). Small blocks of embedded cells were sliced and post-fixed in 2.5% glutaraldehyde for 24 h followed by washing in 0.1 M phosphate buffer for 24 h. Agarose blocks were then incubated in 1% OsO_4_ (in 0.2 M phosphate buffer) for 3 h, washed twice in distilled water and dehydrated using ascending alcohol concentrations (i.e. 25%, 35%, 50%, 70%, 85%, 95%, 100%; each step for 5 min). Dehydrated blocks were incubated in propylenoxide followed by subsequent 20 min incubation in a 1:1 mixture of epon (47.5% glycidether, 26.5% dodenylsuccinic acid anhydride, 24.5% methylnadic anhydride and 1.5% Tris (dimethylaminomethyl) phenol) and propylenoxide. The samples were then incubated in epoxy resin for 1 h at room temperature followed by polymerisation (28 °C for 8 h, 80 °C for 2.5 h and finally at room temperature for 4 h). Ultra-thin sections (70 nm) were mounted on grids for electron microscopy and examined using a Philips CM10 transmission electron microscope as already described^19^.

### Image acquisition

Images of the DAB-stained sections were taken with a Zeiss Axioplan microscope equipped with a 40x objective and an Axio Cam 506 color camera (Zeiss). Images from immunofluorescence labelled sections were taken with a Zeiss LSM 700 laser scanning confocal microscope using ×20, ×40 and ×63 objectives. Images were acquired by averaging 4 scans per area of interest resulting in an image size of 1024 × 1024 pixels. The laser intensity was kept constant for all the samples examined. Captured confocal images were analysed using Adobe Photoshop CS5 and ZEN (Blue edition) 2009 software.

### Statistical analysis

We used one-way ANOVA for comparison of multiple groups within individual experiments and also applied unpaired Student’s *t* test for comparison between two sample groups for the evaluation of significant differences using Graph pad Prism software. Values were expressed as mean ± standard error of mean (SEM) from three independent experiments. For the FRAP and co-localisation experiments, we used the Mann–Whitney *U* test. For both statistical tests, differences between the compared experimental conditions were regarded as significant when *P < 0.05, ***P < 0.0001, while # denotes absence of a significant difference. For the quantification of numbers of autophagosomes (Fig. 2h), large, membrane-bound autophagosomes, filled with the cargoes were counted manually from EM images for 20–30 cells from both wt-VAPB as well as P56S-VAPB cells. Sample sizes of all experiments were determined based upon our experience. No sample was excluded from the analyses. Animals were carefully assigned during collection not randomly, based upon same strain, sex, and age. The replicates numbers are indicated in each figure legend.

## Results

### P56S-VAPB leads to defective autophagy in ALS8

**Fig. 1:**
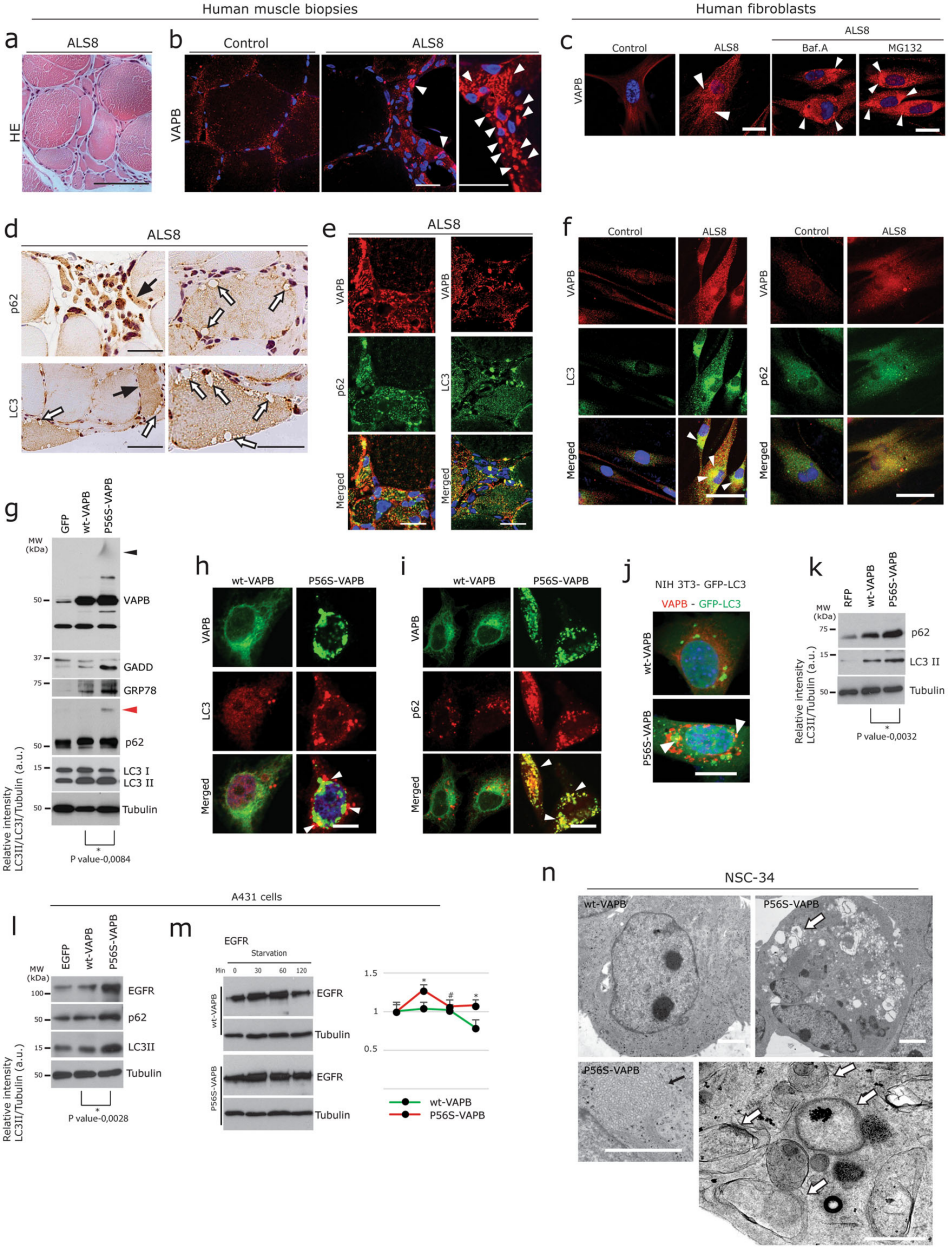
P56S-VAPB leads to defective autophagy in ALS8. **a** Representative H&E stained section from ALS8 index patient muscle biopsy showing typical neurogenic atrophy; presence of angular, flatten and group fibre atrophy. Scale bar: 60 µm. **b** Prominent immunoreactivity of globular VAPB aggregates (arrowheads, right panels) in grouped angular or flattened, atrophic, denervated muscle fibres of ALS8 compared to the normal control (left panel) showing diffuse pattern of sarcomeric VAPB immunolabelling in normal-sized fibres. Scale bars: 70 µm. **c** Immunolabeling of human fibroblasts using VAPB antibody showing small but enormous punctate P56S-VAPB aggregates in ALS8 fibroblast (right panel, white arrowheads), compared to control (left panel). Note the increased formation of globular P56S-VAPB aggregates (arrowheads) in ALS8 fibroblasts, after treatment with proteasome inhibitor MG132 (2 µM) and Bafilomycin A (200 nM) for 4 h. Scale bars: 10 µm. **d** DAB immunohistochemistry performed on ALS8 muscle biopsy showing increased accumulation of autophagy marker p62 and LC3 in atrophic and partially atrophic fibres. Note the numerous large autophagic vacuoles (white arrows) present in hypertrophic fibres. Scale bars: 50 µm. **e** Double immunofluorescence labelling of ALS8 muscle biopsy using antibodies against VAPB and for the autophagy marker p62 and LC3 showing co-localisation of the aggregated p62 and LC3 together with the mutant VAPB aggregates. Scale bars: 25 µm. **f** Punctate P56S-VAPB aggregates co-localised with LC3 and p62 accumulations in ALS8 fibroblasts. Scale bars: 10 µm. **g** Immunoblot analysis in NSC-34 cells transiently transfected either with empty GFP vector, wt-VAPB and P56S-VAPB. Note the increased levels of ER stress, autophagy markers as well as presence of gel top smear aggregate of P56S-VAPB (black arrowhead) and aggregated p62 (red arrowhead) in P56S-VAPB expressing cells. Corresponding densitometric data are shown at the bottom; representing the relative band intensity of LC3-II/LC3-I, normalised with tubulin levels. Graph pad prism, unpaired Student’s *t* test for comparison between two sample groups. Values were expressed as mean ± standard error of mean (SEM) from three independent experiments. The asterisks denote significant differences (*p < 0.05). **h**, **i** Co-labelling of wt-VAPB and P56S-VAPB with LC3 (**h**) and with p62 (**i**). Note that the globular accumulations of both LC3 and p62 and their co-localisation with P56S aggregates. Scale bar: 10 µm. **j**, **k** Increased accumulation of autophagosomes (white arrowheads) in the stable autophagy reporter cell line NIH3T3-GFP transfected with P56S-VAPB. Green: GFP-LC3; red: VAPB; scale bar: 10 µm (**j**). Immunoblot analysis showing increased levels of both p62 and LC3 in cells transfected with P56S-VAPB (**k**). Unpaired Student’s *t* test for comparison between two sample groups. Values were expressed as mean ± standard error of mean (SEM) from three independent experiments. The asterisks denote significant differences (*p < 0.05). **l** Immunoblot analysis of A431 cells transfected with EGFP, wt-VAPB and P56S-VAPB showing the increased levels of the autophagy markers EGFR, p62 and LC3 in P56S-VAPB expressing cells. Unpaired Student’s t-test for comparison between two sample groups. Values were expressed as mean ± standard error of mean (SEM) from three independent experiments. The asterisks denote significant differences (*p < 0.05). **m** A431 cells were transfected as described above. 24 h later, transfected cells were processed for the EGFR degradation assay (see Methods) and analysed by immunoblotting with the EGFR antibody. Note the delayed EGFR degradation in P56S-VAPB expressing cells. Right panel: quantification of immunoblots analysis. Graph pad prism, unpaired Student’s *t* test for comparison between two sample groups. Values were expressed as mean ± standard error of mean (SEM) from three independent experiments. The asterisks denote significant differences (*p < 0.05), while # denotes absence of a significant difference. **n** NSC-34 cells overexpressing wt-VAPB and P56S-VAPB were fixed with 2.5% buffered glutaraldehyde and processed for EM. Several membrane-bound vacuolar structures (white arrows) often containing cargoes and probably derived from the ER in a representative P56S-VAPB-expressing cell compared to cells expressing wt-VAPB control. Higher magnification showing the ER membrane proliferation into tubular structures (black arrow; lower left panel), and large autophagic vacuoles often filled with cargoes (white arrow; lower panel) in representative P56S-VAPB expressing cells. Scale bars: 1 µm (upper panel), 0.5 µm (lower panel).

Over expressed P56S-VAPB forms ER-associated aggregates in cells, hiPSC MNs (Supplementary Fig. 1a, b) as well as in the P56S-VAPB transgenic mouse models (Supplementary Fig. 1c); in contrast, P56S-VAPB inclusions were absent in induced pluripotent stem cell (iPSC)-derived MNs of ALS8 patients^43^. Autopsy tissues from ALS8 patients are not available so far to address the toxic gain of function of P56S-VAPB, however, for current study, we could use muscle biopsies obtained from an index ALS8 patient of European origin^5^ and from his affected mother. H&E staining of the index patients’ muscle biopsy and the routine histology from his affected mother showed severe neurogenic grouped fibre atrophy (Fig. 1a and Supplementary Fig. 1d), associated with increased immunoreactivity for several ER chaperones and proteotoxicity markers (Supplementary Fig. 1e) in partially atrophic and atrophic muscle fibres of the index patient.

Using our custom made VAPB antisera^31–33^, we found accumulation of globular aggregates of VAPB in denervated muscle fibres (Fig. 1b, arrowheads). Normal-sized innervated muscle fibres in this biopsy as well as control biopsies showed mild to moderate sarcoplasmic VAPB immunoreactivity (Fig. 1b). Skin fibroblasts obtained from this ALS8 patient revealed smaller prominent punctate VAPB aggregates (Fig. 1c), which formed larger globular aggregates when challenged with proteasome or autophagy inhibitors (Fig. 1c; arrowheads right panel). Western blot analysis confirmed the presence of SDS-insoluble gel top VAPB aggregates in ALS8 fibroblast (Supplementary Fig. 1f)

Since VAPB protein is crucial for autophagy^14,20^, we used the ALS8 muscle biopsy and the patient-derived fibroblasts to understand if aggregated P56S-VAPB affects the autophagy pathway.

DAB immunohistochemistry using antibodies against autophagic markers LC3 and p62 revealed their accumulations in degenerating fibres containing large autophagic vacuoles (Fig. 1d; arrows). Immunofluorescence showed even more prominent accumulations of large LC3 and p62 puncta, which were specifically co-localised with globular VAPB aggregates (Fig. 1e). Consistent with these findings, ALS8 fibroblasts also showed co-localisation of accumulated LC3 and p62 together with P56S-VAPB aggregates (Fig. 1f; arrowheads).

P56S-VAPB aggregates sequestering both LC3 and p62 were indicative of altered autophagy. Thus we next analysed the autophagy status in several cell lines expressing P56S-VAPB tagged with GFP following current guidelines^44–47^. Western blot analysis showed LC3 II and p62 were elevated together with the ER stress markers GRP78 both in P56S-VAPB transfected cells (Fig. 1g and Supplementary Fig. 1g) and in ALS8 fibroblasts (Supplementary Fig. 1f). Interestingly, consistent with the appearance of SDS insoluble VAPB smears (black arrowhead, Fig. 1g) in P56S-VAPB expressing cells, p62 protein was also aggregated at the gel top (red arrowhead, Fig. 1g). In line with this LC3 and p62 positive autophagosomes were found to be abnormally accumulated and sequestered with P56S-VAPB aggregates (Fig. 1h, i and Supplementary Fig. 1h, i). Similarly, a consistent pattern of accumulated LC3 by immunofluorescence analysis (Fig. 1j), confirmed by Western blot studies (Fig. 1k) was observed in NIH3T3 fibroblasts stably expressing GFP-LC3^35,42^. Furthermore, we applied a widely used method to analyse the internalization and degradation of the EGF-EGFR complex in A431 cells that express high EGFR levels which is exclusively degraded via autophagy^35,42,48^. Here again, expression of P56S-VAPB led to the accumulations of EGFR together with p62 and LC3 (Fig. 1l). The expression of wt or P56S- VAPB did not impair EGFR internalization after 10 min of EGF stimulation (not shown). However, P56S-VAPB, in contrast to wt-VAPB, impeded time-dependent degradation of EGFR (Fig. 1m). Defective autophagy was further characterised by ultra-structural analysis of P56S-VAPB-transfected cells, showing prominent accumulations of numerous autophagic vacuoles (white arrows) which also sequestered cellular components/cargoes (Fig. 1n). These changes were accompanied by the cytoplasmic accumulation of tubular structures (black arrow), probably reflecting ER proliferation (Fig. 1n).

### P56S-VAPB alters both early and late steps of autophagy

**Fig. 2:**
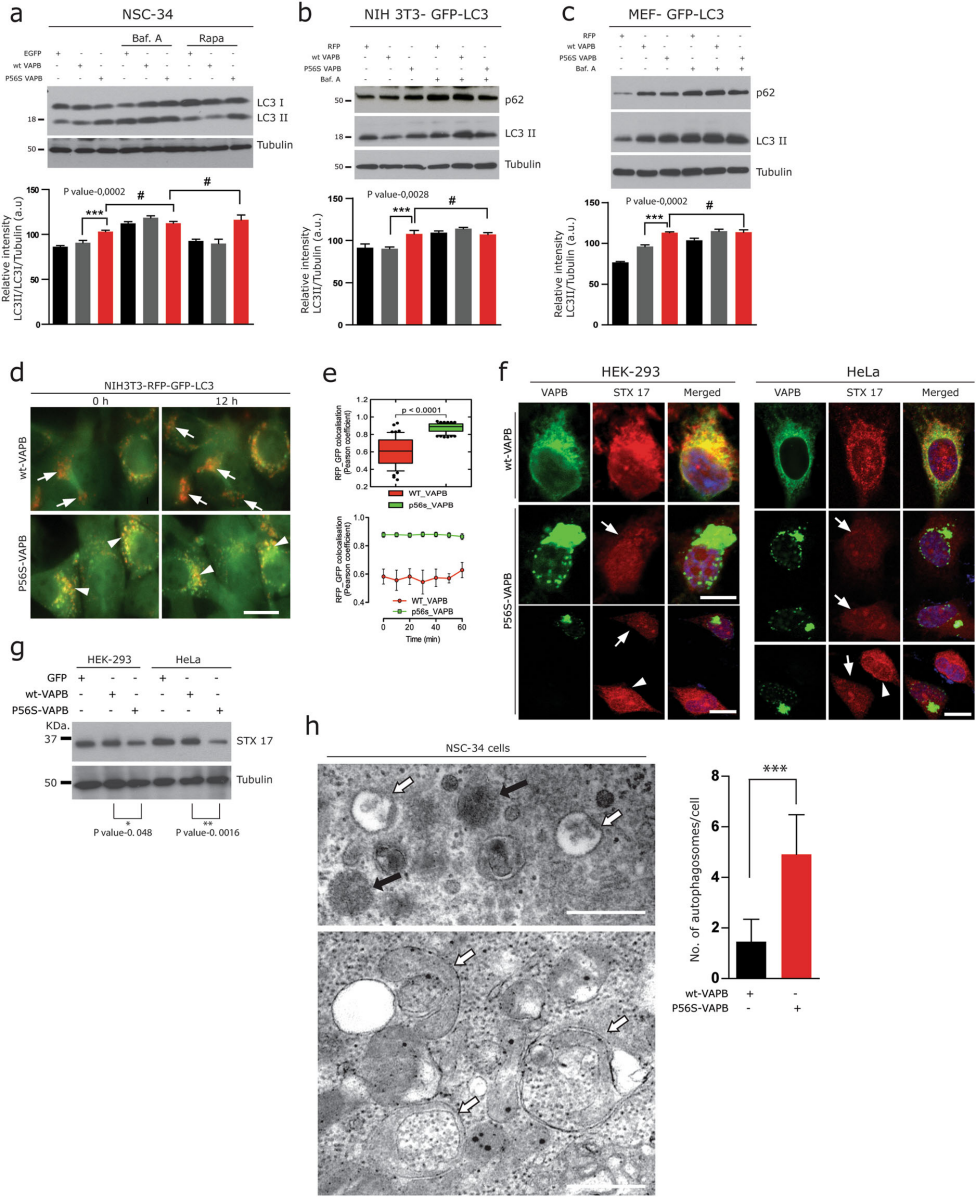
P56S-VAPB alters late step of autophagy. **a**–**c** Immunoblot analysis of transiently transfected control GFP/RFP, wt-VAPB and P56S-VAPB; in NSC-34 cells (**a**), NIH3T3-GFP-LC3 fibroblasts (**b**) and of the Primary fibroblasts isolated from autophagy reporter GFP-LC3 transgenic mice (MEF-GFP-LC3) (**c**), and then additionally treated with the autophagy inhibitor Bafilomycin A (2 µM) for 2 h to inhibit autophagy followed by treatment of Rapamycin (2 µM) for 4 h to accelerate autophagy (**a**) or Bafilomycin A (2 µM) alone (**b**, **c**). Note the unchanged LC3-II levels in P56S-VAPB transfected cells after Rapamycin or Bafilomycin A treatment. Corresponding densitometric data are shown at the bottom; where the numbers represent the relative band intensity of LC3-II/LC3-I, normalised with tubulin levels. Graph pad prism, unpaired Student’s *t* test for comparison between two sample groups. Values were expressed as mean ± standard error of mean (SEM) from three independent experiments. The asterisks denote significant differences (*p < 0.05, ***p < 0.001), while # denotes absence of a significant difference. **d**, **e** NIH3T3 fibroblasts stably expressing RFP-GFP-LC3 was transfected either with HA tagged wt-VAPB or P56S-VAPB. In all, 24 h later the fusion of autophagosomes with lysosomes was measured by live cell imaging for additional 12 h (**d**), the rate of autophagosome fusion reflected by the Pearson coefficient (green/red fluorescence ratio) at each time point indicated (**e**). Values are represented as means ± S.E.M. of triplicate experiments *p ≪ 0.0001. **f** Co-labelling of wt-VAPB and P56S-VAPB transfected HEK-293 as well as HeLa cells with STX17, showing its marked reduction (arrows) in cells harbouring P56S-VAPB aggregates. Scale bar: 10 µm. **g** Immunoblot analysis of STX17 proteins in HEK-293 as well as HeLa cells, transiently transfected with control GFP, wt-VAPB and P56S-VAPB. Note the significantly reduced levels of STX17 in P56S-VAPB overexpressing cells. Unpaired Student’s *t* test for comparison between two sample groups. Values were expressed as mean ± standard error of mean (SEM) from three independent experiments. The asterisks denote significant differences (*p < 0.05). **h** EM analysis of P56S-VAPB transfected NSC-34 cells showing massive accumulation of autophagosomes (white arrows, quantification) in the vicinity of several lysosomes (black arrows). For the quantification of autophagosomes, numbers of large, membrane-bound autophagosomes, filled with the cargoes were counted manually from 20 to 30 cells from both the wt-VAPB as well as P56S-VAPB. The asterisks denote significant differences (***p < 0.001). Scale bars: 1 µm.

Aggregated p62 at the gel top, co-localisation of LC3 and p62 with P56S-VAPB aggregates and increased LC3-II in Western blots (Fig. 1) were all indicative of blocked autophagic flux. However, to further rule out that these effects were not due to increased autophagic activity, we next systematically monitored autophagic flux^45,47^. Compromised degradation of accumulated LC3II due to P56S-VAPB was confirmed by Western blot analysis in NSC-34 cells, where autophagy induction (Rapamycin) or inhibition (Bafilomycin A) only marginally, if at all, affected p62 and LC3-II levels in P56S-VAPB expressing cells compared to wt-VAPB (Fig. 2a). Consistently in GFP-LC3 reporter lines and in primary mouse embryonic fibroblasts, the absence of a significant increase in LC3-II and p62 protein levels even after Bafilomycin A treatment (Fig. 2b, c) further confirmed impaired clearance of accumulated autophagosomes.

A possible scenario underlying the above phenomenon might be the fusion defects of autophagosomes to lysosomes in P56S-VAPB expressing cells. To test this possibility, we monitored the fusion of autophagosomes to lysosomes using previously described NIH3T3- mCherry-GFP-LC3 cells^49^. As the GFP signal is lost upon degradation in the acidic environment of lysosomes, loss of GFP signal relative to mCherry signal is a read out of autophagosome fusion with lysosomes^49^. Consistent with this notion, live cell imaging showed that GFP signal was relatively preserved in mCherry-GFP-LC3 cells that also expressed P56S-VAPB, suggesting that the fusion of autophagosomes with lysosomes was impaired by P56S-VAPB (Fig. 2d, e). In contrast, we observed both a clear reduction of GFP fluorescence and RFP-GFP co-localisation in nearly all wt-VAPB expressing cells (Fig. 2d, e). To further support this conclusion, we analysed the SNARE protein syntaxin 17 (STX17) which is essential for the fusion of lipid bilayers and required for autophagosome-lysosome fusion^50^. Using Immunofluorescence analysis, we observed that STX17 protein immunolabelling were markedly reduced in HEK-293 as well as in HeLa cells harbouring P56S-VAPB aggregates (Fig. 2f, arrows). Consistent with this findings Western blot analysis of the above cell lysates showed a significant decrease in STX17 protein levels in P56S-VAPB overexpressing cells compared to the wt-VAPB and control GFP overexpressing cells (Fig. 2g, quantification below).

Finally, electron microscopy performed on NSC-34 cells revealed that, consistent with impaired fusion, massive accumulation of autophagosomes with cargoes (white arrows) were found in the vicinity of lysosomes (black arrows), indicative of impairment of their fusion (Fig. 2h, quantification of the accumulated autophagosomes). Taken together, our findings demonstrate that P56S-VAPB impairs the autophagic degradation due to incomplete clearance of autophagosomes (i.e., late-stage autophagy).

Recently, Zhao et al. reported that P56S-VAPB mutation reduces the ULK1/FIP200 interaction and impairs autophagy at an early stage, namely autophagosome biogenesis^14^. Therefore, as a next logical step, we also analysed the effect of P56S-VAPB on early steps of autophagy. We focussed on ULK1 as well as on ATG5-ATG12, because the ATG5- ATG12 containing complex is essential for membrane expansion and completion of the autophagosome^51–53^

**Fig. 3:**
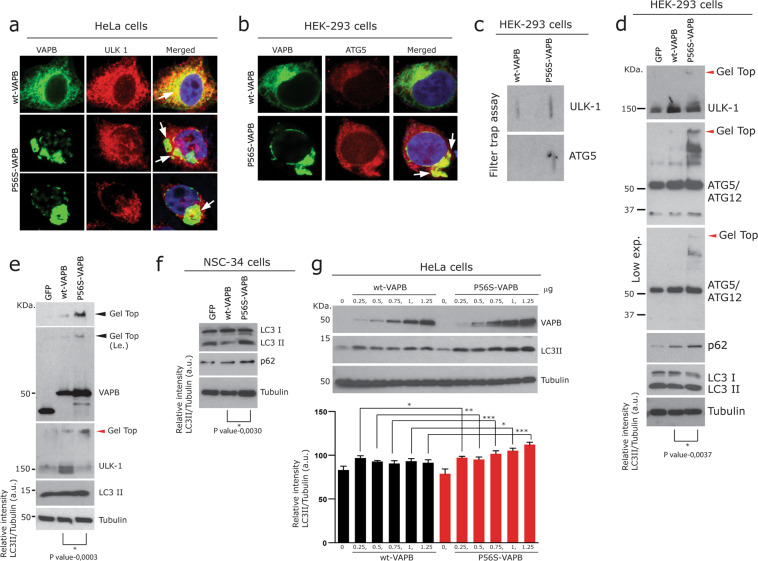
P56S-VAPB alters early steps of autophagy. **a** Co-labelling of wt-VAPB and P56S-VAPB transfected HeLa cells with ULK1 showing co-localisation with wt-VAPB. Note the reduction as well as the sequestration of ULK1 (arrows; merged middle and lower images) with P56S-VAPB aggregates. Scale bar: 10 µm. **b** Sequestration of ATG5-ATG12 (arrows) with P56S-VAPB aggregates in HEK-293 cells Scale bar: 10 µm. **c** Filter trap assay of the above transfected cells, showing SDS resistant aggregates of ULK1 and ATG5-ATG12 in P56S-VAPB expressing cells. **d**, **e** Immunoblot analysis showing gel top aggregation (red arrowheads) as well as reduced soluble levels of ULK1 together with increased levels of LC3-II in P56S-VAPB overexpressing HEK-293 cells (**d**) and in HeLa cells (**e**). Note the gel top aggregation (red arrowheads) of ATG5-ATG12 complex in P56S-VAPB overexpressing cells as well as increased levels of ULK1 in wt-VAPB overexpressing cells. Unpaired Student’s *t* test for comparison between two sample groups Values were expressed as mean ± standard error of mean (SEM) from three independent experiments. The asterisks denote significant differences (*p < 0.05, ***p < 0.001). **f** Immunoblot analysis of NSC-34 cells showing increased levels of LC3-II and p62 in P56S-VAPB expressing NSC-34 motor neuron-like cells. Unpaired Student’s *t* test for comparison between two sample groups Values were expressed as mean ± standard error of mean (SEM) from three independent experiments. The asterisks denote significant differences (*p < 0.05). **g** Immunoblot analysis (quantification below) showing concentration dependent increased levels of LC3-II and p62 in P56S-VAPB overexpressing cells compared to the wt-VAPB expressing HeLa cells. Graph pad prism, One-way ANOVA, values were expressed as mean ± standard error of mean (SEM) from three independent experiments. The asterisks denote significant differences (*p < 0.05, ***p < 0.001).

While both ULK1 and ATG5-ATG12 proteins were distributed throughout the cell, largely co-distributing with wt-VAPB (Fig. 3a, b); however, they both sequestered with P56S-VAPB aggregates in P56S-VAPB expressing cells (Fig. 3a, b arrows). Filter trap assays further confirms the presence of SDS resistant aggregates of both of these proteins in P56S-VAPB expressing cells (Fig. 3c). Consistent with this, Western blots indicated that ULK1 protein levels were elevated in wt-VAPB expressing cells (Fig. 3d), whereas in P56S-VAPB expressing cells, fractions of ULK1 protein remained at the gel-top as an aggregated form (Fig. 3d, e; red arrowhead), thus resulting in reduced amounts of its soluble form (Fig. 3d, e). Similar to ULK1 and consistent with the filter trap assay, ATG5-ATG12 protein complex (actual molecular weight = 55 kDa) was also found to be aggregated at the gel-top (Fig. 3d). Even though such alterations of crucial autophagy-associated proteins are consistent with defects in early phases of autophagy^14^; increased levels of LC3-II in the same samples (Fig. 3d, e) together with our earlier observations (Fig. 2) suggest defects in late-stage autophagy. We used multiple cell lines expressing either the wt or P56S-VAPB (Figs. 2, 3d–g, and Supplementary Fig. 1j) and in most of them P56S-VAPB induced both the reduction of soluble form / aggregation of ULK1 at the gel top as well as increased levels of p62/LC3-II. Based on these observations, we concluded that P56S-VAPB alters both late and early steps of autophagy simultaneously.

### Autophagy defects in P56S-VAPB transgenic mice

**Fig. 4:**
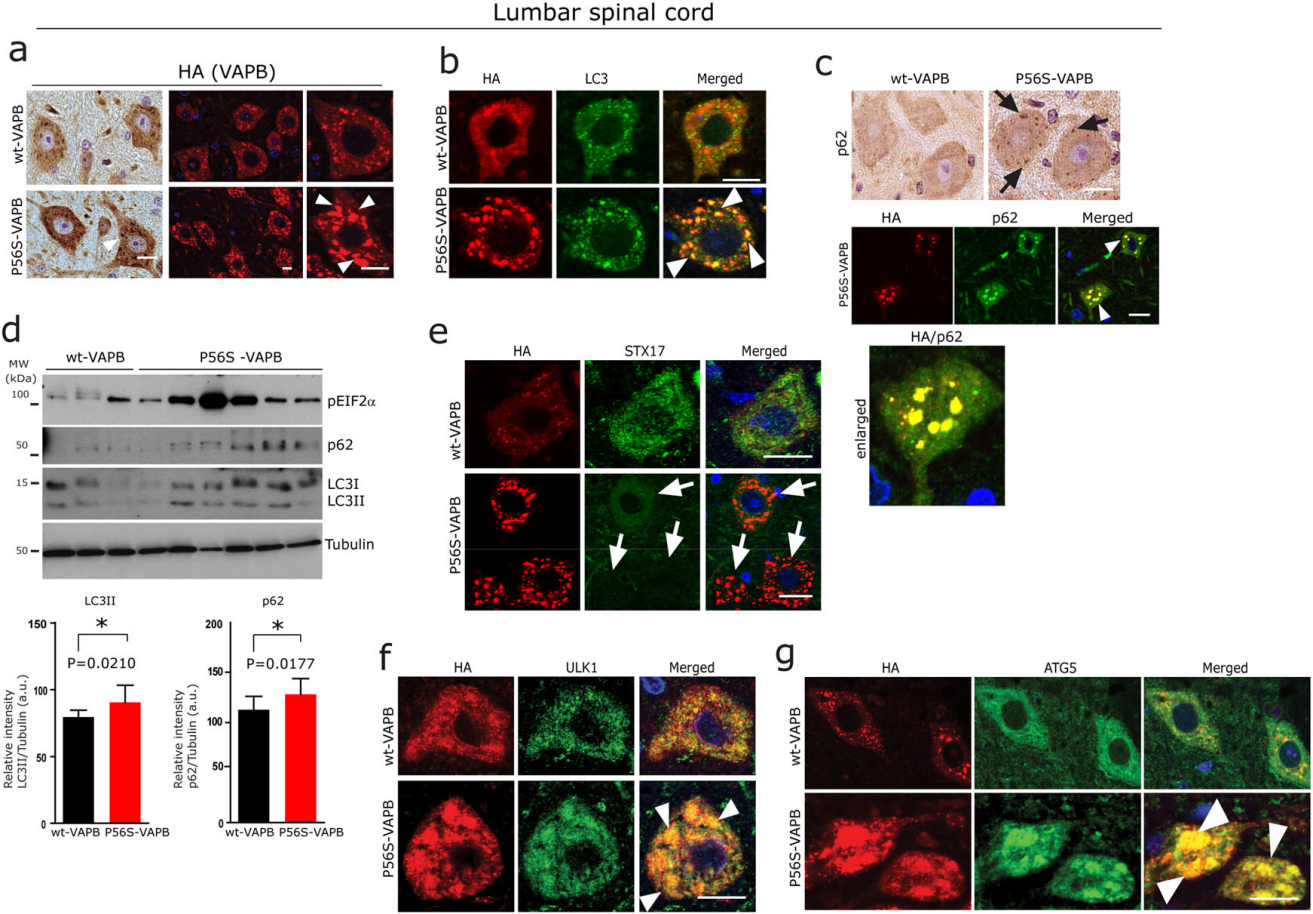
Autophagy defects in P56S-VAPB transgenic mice. **a** Representative DAB immunohistochemistry (Left panel) and immunofluorescence staining (right panel) using HA antibody in lumbar spinal cord α-MNs of wt and P56S-VAPB tg mice, showing globular aggregates in the α-MNs (arrowheads) of P56S-VAPB tg mice. Scale bars: 15 µm. **b**, **c** Double immunofluorescence labelling for HA and for the autophagy marker LC3 (**b**) and p62 lower panel showing co-localisation (arrowheads) with P56S-VAPB aggregates (**c**), and DAB immunohistochemistry showing globular accumulations of p62 (arrows, upper panel) (**c**) in lumbar spinal cord α-MNs, Scale bars: 15 µm. **d** Immunoblot analysis of lumbar spinal cord homogenates from P56S-VAPB (*n* = 6, male, 200 days) and their age-matched wt-VAPB mice (*n* = 3, male, 201 days), showing an overall increased level of ER stress markers pIF2α and autophagy markers p62 and LC3 in P56S-VAPB tg mice. Corresponding densitometric data are shown at the bottom; representing the average relative band intensity from *n* = 3 wt-VAPB and *n* = 6 P56S-VAPB of p62, LC3-II/LC3-I normalised with tubulin levels. Unpaired Student’s *t* test for comparison between these two sample groups (average, *n* = 3 wt-VAPB mice and average, *n* = 6 P56S-VAPB mice) Values were expressed as mean ± standard error of mean (SEM) from three independent blots. The asterisks (*) denote significant differences (*p < 0.05). **e**–**g** Double immunofluorescence labelling for HA and for the SNARE protein STX17 (**e**), early autophagy proteins ULK1 (**f**) and ATG5-ATG12 (**g**) showing their co-localisation (arrowheads) with P56S-VAPB aggregates in lumbar spinal cord α-MNs. Note the loss of STX17 immunoreactivity (arrows in **e**) in α-MNs harbouring P56S-VAPB aggregates in lumbar spinal cord of P56S-VAPB mice. Scale bars: 15 µm.

Prompted by the above findings we next examined autophagy markers in transgenic mice expressing P56S-mutant human *VAPB* that develop VAPB aggregates both in lumbar spinal cord α-MNs as well as in cortical neurons accompanied by ER stress^15,22^ (Fig. 4a and Supplementary Fig. 2c–f). Both lumbar spinal cord α-MNs (Fig. 4b, c) and cortical neurons (Supplementary Fig. 2e) consistently showed co-localisation of LC3 and p62 together with globular P56S-VAPB aggregates. In line with the above observations in cell culture models (Figs. 2 and 3), we also found significantly reduced immunoreactivity of STX17 (Fig. 4e) and sequestration of early autophagy proteins ULK1, ATG5-ATG12 together with the globular P56S-VAPB aggregates in the lumbar spinal cord α-MNs of P56S-VAPB mice (Fig. 4f, g). Consistent with this, Western blot analysis of lumbar spinal cord lysates and the total brain lysates revealed increased levels of p62 as well as LC3 together with markedly increased UPR (Fig. 4d and Supplementary Fig. 2h). Interestingly, here again p62 appeared as SDS-insoluble aggregates at the gel-top in the immunoblots of total brain lysates of these mice (Supplementary Fig. 2h, red arrowhead). These in vivo results together with the cell culture observations establish that autophagy is altered at multiple stages, leading to neuronal and axonal degeneration of both cortical as well as spinal cord α-MNs (Supplementary Fig. 2a, f, g).

### P56S-VAPB aggregation leads to altered distribution of other fALS-associated RBP

**Fig. 5:**
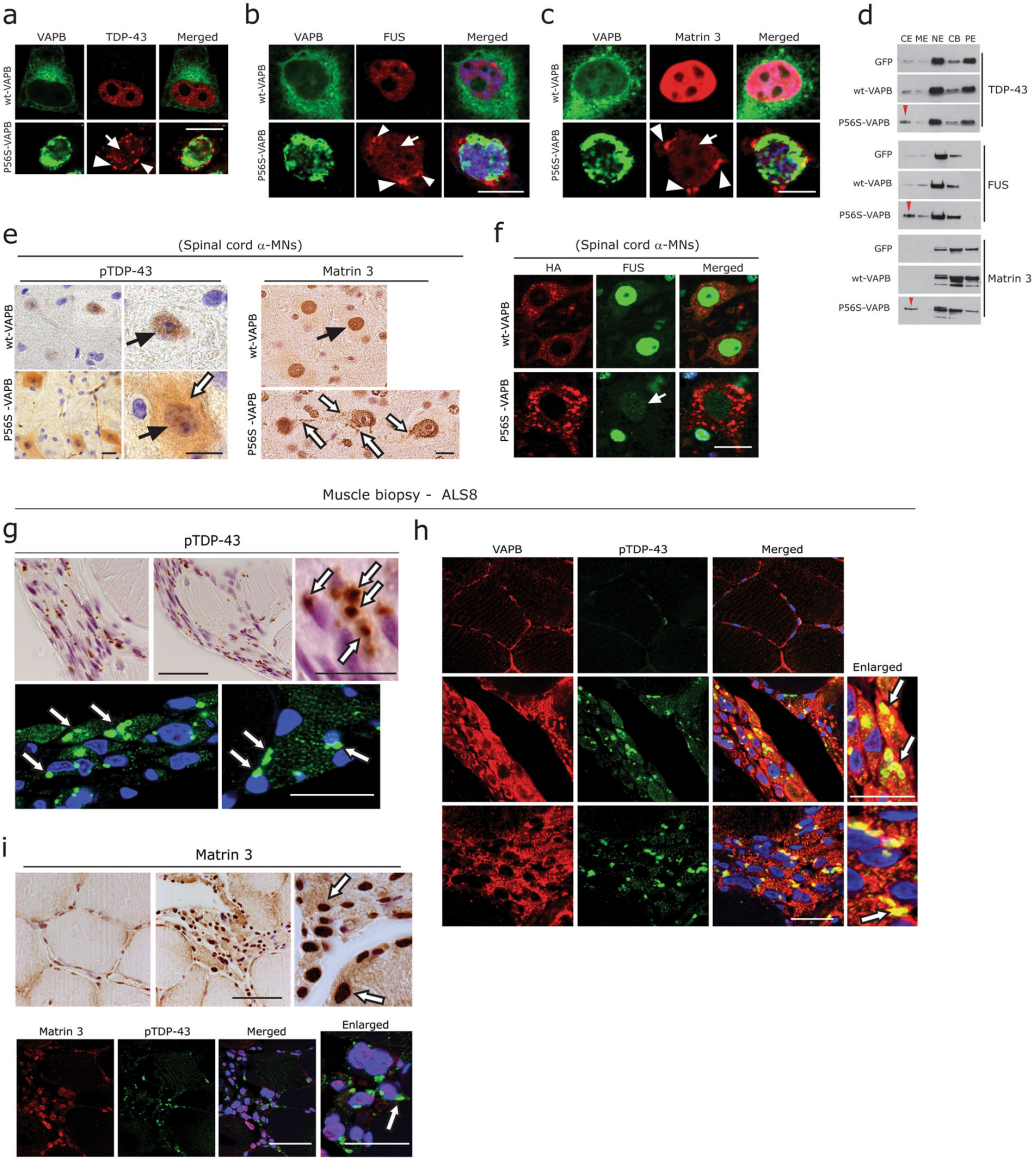
P56S-VAPB and altered distribution of other fALS-associated RBP. **a**–**c** Co-labelling of HeLa cells expressing wt-VAPB and P56S-VAPB with antibodies against TDP43 (**a**) with FUS (**b**) and with Matrin 3 (**c**). Note that the cytoplasmic aggregation (arrowheads) and reduced nuclear immunoreactivity (arrows) of these RBPs in P56S-VAPB expressing cells. Scale bar: 10 µm. **d** Immunoblot analysis of the subcellular fractions obtained from HeLa cell expressing wt-VAPB and P56S-VAPB. Note the increased levels of TDP-43, FUS and of Matrin 3 (red arrowheads) in cytoplasmic extract (CE). ME membrane extract, NE nuclear extract, CB chromatin bound, PE cytoskeleton. **e** DAB immunohistochemistry using antibodies against pTDP-43 and Matrin 3, showing altered nuclear immunoreactivity (black arrows) and diffuse albeit increased cytoplasmic immunoreactivity (white arrows) in lumbar spinal cord α-MNs of P56S-VAPB tg mice. Scale bars: 15 µm. **f** Double immunofluorescence labelling for HA and for FUS antibodies showing reduced labelling of FUS (arrow) in lumbar spinal cord α-MNs of P56S-VAPB tg mice. Scale bars: 15 µm. **g** Prominent and globular pTDP-43 immunoreactivity (white arrows- upper panel; DAB, lower panel; immunofluorescence) of grouped angular or flattened, atrophic, denervated muscle fibres in the ALS8 biopsy. Scale bars: 80 µm. **h** Co-localisation of globular and aggregated pTDP43 with VAPB aggregates (white arrows) in the same biopsy. Scale bars: 60 µm. **i** DAB immunohistochemistry of Matrin 3, showing increased immunoreactivity of the myonuclei (arrows) and mildly diffuse saromeric accumulations. Note the basal myonuclei immunoreactivity of Matrin 3 in non-atrophic normal-sized fibres (left panel). Co-immunolabelling (lower panel) with pTDP-43 antibody showed no co-localisation between Matrin 3 accumulations and pTDP-43 aggregates. Scale bars: 80 µm.

Efficient turnover of several fALS-associated RBPs such as TDP-43, FUS and of other RNA SGs is regulated by UPS and autophagy^54,55^. Similarly, alterations of RBP homeostasis can directly affect proteostasis (reviewed in Walsh et al.^56^). Since P56S-VAPB aggregates were associated with impaired autophagy, we asked whether P56S-VAPB-mediated toxicity is also linked to the altered distribution RBPs, in particular the key ALS proteins TDP43, FUS and Matrin 3. These proteins normally reside in the nucleus; in ALS, they often accumulate in the cytoplasm of affected neurons^1,26,29^. We found that P56S-VAPB aggregates are associated with loss of nuclear localisation and cytoplasmic accumulation of above mentioned RBPs (Fig. 5a–c) compared to the wt-VAPB transfected cells, where they remained in the nucleus (Fig. 5a–c; upper panels). In many instances, P56S-VAPB was partially co-localised with some of these cytoplasmic RBP aggregates (Fig. 5a–c). In line with this, subcellular fractionation performed on P56S-VAPB expressing cells further confirmed the elevated protein levels of TDP-43, FUS and Matrin 3 in the cytoplasmic fractions (Fig. 5d, red arrowheads). Accordingly, there was mild to moderate loss of nuclear localisation combined with diffuse cytoplasmic accumulation of pTDP-43 and Matrin 3 in α-MNs of P56S-VAPB compared to wt-VAPB Tg mice (Fig. 5e). Even though cytoplasmic FUS accumulations were rather rare, loss of nuclear FUS was prominent in the α-MNs of the P56S-VAPB mice (Fig. 5f). Prompted by these observations, we further examined the ALS8 patient muscle biopsy. Similar to the characteristic pTDP-43 aggregates found in sporadic and familial ALS α-MNs, degenerating muscle fibres in this muscle biopsy displayed accumulations of large globular pTDP-43 aggregates (Fig. 5g). pTDP-43 aggregates can actually be seen in atrophic and partially atrophic fibres of many sALS and fALS autopsy cases^57^ (Supplementary Fig. 3d). Interestingly, these abundant pTDP-43 aggregates were also found to be co-localised with globular VAPB aggregates (Fig. 5h, arrows, right panels). As expected, Matrin 3 immunoreactivity of myonuclei of degenerating muscle fibres were elevated (Fig. 5i), while the normal-sized fibres showed mild labelling of the myonuclei (Fig. 5i; left panel). A mild diffuse pattern of cytoplasmic Matrin 3 immunoreactivity was also observed in many degenerating fibres (Fig. 5i); however, there were no obvious cytoplasmic aggregates of Matrin 3 in this biopsy and there was no co-localisation with pTDP-43 aggregates (Fig. 5i).

### P56S-VAPB accumulation leads to abnormal stress granule formation

**Fig. 6:**
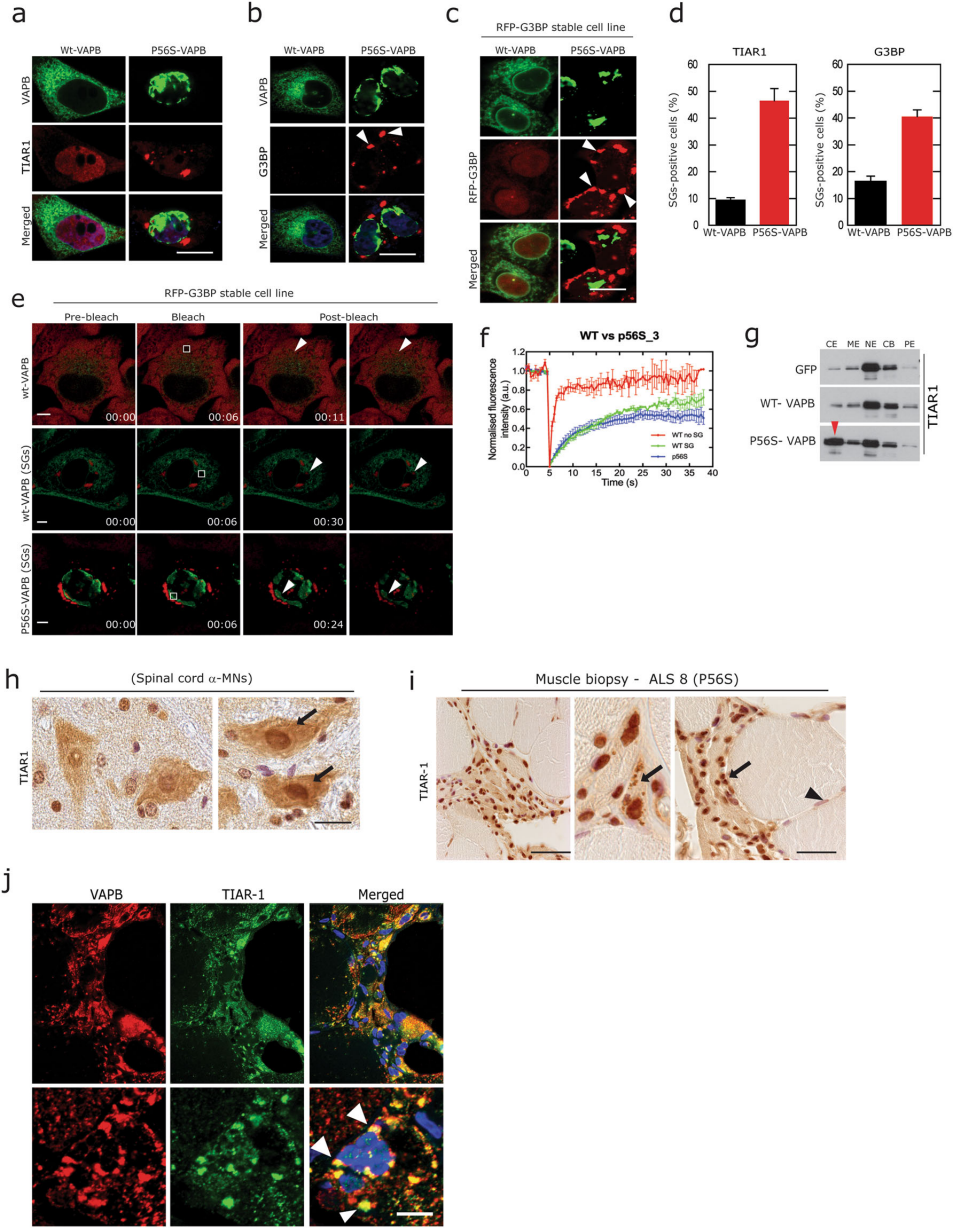
P56S-VAPB leads to abnormal stress granules formation and alters their dynamics. **a**, **b** Co-labelling of HeLa cells expressing wt-VAPB and P56S-VAPB with antibodies against SG markers TIAR1 (**a**) and G3BP (**b**), showing cytoplasmic SG formation (arrowheads) in P56S-VAPB expressing cells. Scale bar: 10 µm. **c** Immunofluorescence analysis of HeLa cells stably expressing SG protein G3BP, showing SG formation in P56S-VAPB transfected cells compared to wt-VAPB transfected cells after 24 h. Scale bar: 10 µm. **d** Quantification of TIAR1 in HeLa cells and G3BP stable cell lines for SGs formation due to overexpression of either the wt-VAPB or P56S-VAPB after 36 h of transfection. **e**, **f** FRAP analysis by live cell imaging on the above cell lines showing reduced recovery of G3BP-SGs in P56S-VAPB transfected cells compared to wt-VAPB transfected cells, Quantifications (**f**). Scale bar: 10 µm. **g** Immunoblot analysis subcellular fractions of HeLa cells expressing wt-VAPB and P56S-VAPB, showing increased levels of TIAR1 in cytoplasmic fraction (red arrowhead, CE) in P56S-VAPB expressing cells. **h**, **i** DAB immunohistochemistry using antibodies against SG marker TIAR1 in lumbar spinal cord α-MNs of wt and P56S-VAPB Tg mice (**h**) and in ALS8 muscle biopsy (**i**), showing altered nuclear immunoreactivity (arrows) and diffuse albeit increased cytoplasmic immunoreactivity (arrows) in both α-MNs of P56S- VAPB tg mice as well as in ALS8 muscle biopsy. (Scale bars: 15 µm in **h**, 60 µm in **i**. **j** Accumulated globular immunoreactive TIAR1 aggregates co-localises with VAPB aggregates (arrowheads) in ALS8 muscle biopsy. Scale bars: 25 µm.

Since aggregation of the above mentioned RBPs mostly proceeds through the SG pathway^2,26,29,58,59^, we next examined SG formation in cells transfected with wt or P56S-VAPB. Consistent with the mis-localisations of many fALS-associated RBPs, we observed cytoplasmic accumulation of TIAR-1 and G3BP SGs in P56S-VAPB expressing cells (Fig. 6a, b). For further validations, we used a well-studied cell line stably expressing the SG protein G3BP^38^. We found that SG formation in P56S-VAPB transfected cells started within 10 h, even before P56S-VAPB aggregates were formed (15 h after transfection). These aggregates increased in number and size at 24 h (Fig. 6c, d). We could not observe any SG formation in cells expressing wt-VAPB during the same period of time, however at later time (36 h) we could observe minor SGs in few cells (Fig. 6d). Considering that SGs are highly dynamic structures and that persistent stress and misfolded protein aggregation lead to prolonged SG stability and aggregation^38,58,60^, we next studied the dynamic properties of SGs formed by P56S-VAPB using fluorescence recovery after photobleaching (FRAP) analysis to measures the relative kinetics of SGs over the time course of the experiment^38,58,60^. FRAP analysis of these G3BP-SGs showed a slower recovery in P56S-VAPB expressing cells compared rare SGs observed in cells expressing wt-VAPB (Fig. 6e, f). The persistent accumulations of these SGs were further confirmed by subcellular fractionation showing that the SG protein TIAR1 accumulated in the cytoplasmic fraction (Fig. 6g) in P56S-VAPB expressing cells. In line with these cell culture observations, we observed an increased albeit diffuse immunoreactivity of TIAR1 in lumbar spinal cords α-MNs of P56S Tg mice (Fig. 6h). Finally, we observed an enhanced immunoreactivity of large granular TIAR-1-positive SGs in ALS8 patient muscle biopsy (Fig. 6i, arrows). Interestingly, these SGs were co-localised with VAPB aggregates (Fig. 6j, arrowheads). In summary, consistent with the deregulation of RBPs, the robust formation of SGs is a predominant feature of P56S-VAPB-driven pathology.

## Discussion

Altered RBPs homeostasis and defective proteostasis are key mechanisms triggering neurodegeneration in ALS^1,26^. In the present study we asked if P56S-VAPB is involved in these processes to initiates pathological cascades leading to neurodegeneration.

We found accumulations of p62 and LC3 protein and of their sequestration with P56S-VAPB aggregates in various cell culture models, P56S-VAPB mouse, ALS8 patient fibroblast and muscle biopsy (Figs. 1–4). Furthermore, alterations in autophagy flux were confirmed in multiple cell lines (Figs. 1–3). Consistent with this, we found numerous autophagic vacuoles associated with ubiquitin-positive accumulations in ALS8 patient muscle biopsy (Fig. 1). These results are in line with recent reports of P56S-VAPB accumulations in the autophagosome and with the ubiquitin-positive inclusions in MNs of ALS8 knock-in mice that were accompanied by increased levels of P62/SQSTM1 and LC3^24^. Of note in line with the recently described role of VAPB in ER-phagy^61^, we found proliferated, expanded ER both in P56S-expressing cells and in the P56S-VAPB patient muscle biopsy (Fig. 1 and Supplementary Fig. 3e). Thus, it appears reasonable to propose that ER-assisted autophagy dysfunction is an early, central step of P56S-VAPB-mediated ALS pathology. In this context it is important to mention that apart from VAPB, mutations, several ER proteins including SigR1, SIL1, HSPB1, HSPB8 and HSJ1 cause defects in ER structure accompanied by autophagy impairment and lead to familial neurodegenerative disorders including motor neuron diseases^62–68^. In sALS, the above wild-type proteins accumulate in surviving MNs in sALS and might serve protective functions, as has been shown for SigR1 and SIL1^19,69,70^.

VAPB interacts with ATG proteins and also facilitate the recruitment and stabilisation of ULK1 and FIP200 puncta at the ER, which is crucial for autophagosome formation^14^. Loss of VAPB and PTPIP51 (ER-mitochondria tethering complex) loosens the ER-mitochondria contacts and induces autophagic flux, whereas overexpression of wt-VAPB and PTPIP51 tightens the ER-mitochondria contacts and thus impairs autophagy^20,21^. Thus, confirming normal VAPB is decisive for autophagy process. Consistent with these reports, reduction of ULK1 protein levels as well as co-aggregation of ULK1 and ATG5-ATG12 together with P56S-VAPB aggregates depicted in Fig. 3 and in α-MNs of P56S-VAPB mice (Fig. 4) suggests probably an alteration of the initial phase of autophagy. However, the concomitant appearance of significantly increased levels of LC3-II in multiple cell lines overexpressing P56S-VAPB and detailed analysis of autophagic flux using autophagy reporter cell lines confirmed that P56S-VAPB also impairs the fusion of autophagosomes to lysosomes, which is a late autophagy step (Figs. 2 and 3). In this context it is important to note that besides orchestrating initial steps of autophagy, ULK1 can also regulate late steps of autophagy by promoting autophagosome to lysosome fusion^71^.

RBPs including many SGs are particularly susceptible to aggregation due to the presence of both RNA-binding domains and prion-like domains, which contribute strongly to aggregate formation under stressful stimuli including chronic autophagy impairment^72,73^. In addition, loss of dynamic nature of these SGs together with their impaired clearance could results in the formation of aberrant and persistent pathological inclusions^2,38,58,74–76^. Notably, P56S-VAPB mice show signs of TDP-43 pathology^23^. Furthermore, the expression of P56S-VAPB enhanced TDP-43-induced neuronal cell death in vitro, whereas the expression of wt-VAPB attenuated neuronal death^77^. Consistent with this, we found that P56S-VAPB leads to neuronal cell death in iPSC-derived MNs (Supplementary Fig. 1k) and a loss of nuclear localisation and cytoplasmic aggregation of fALS-associated RBPs such as FUS, TDP-43 and Matrin 3 and that of SGs in cultured cells, P56S-VAPB mice, and in ALS8 patient muscle fibres (Figs. 5 and 6). Additionally, supporting the notion that RBP homeostasis largely relies on efficient autophagy, here we confirmed that chemical inhibition of autophagy leads to extra-nuclear localisation of the majority of fALS-associated RBPs (Supplementary Fig. 3) similar to the autophagy defects mediated by P56S-VAPB.

The ALS8 patient muscle biopsy presented can be regarded as a missing piece of the puzzle. On one hand, this is valuable human tissue, because α-MNs from P56S-VAPB patients could not be examined so far because of the lack of autopsy cases. On the other hand, the considerable build-up of pTDP-43 in ALS8 muscle fibres associated with VAPB aggregates is suggestive of primary muscle involvement. This is in agreement with the emerging concept that skeletal muscle pathologies in MNDs are more than just a bystander and can actually contribute to MN degeneration (reviewed in refs. ^33,78^). Such primary muscle fibre damage is also suggested by the presence of characteristic pTDP-43 aggregates in sALS muscle biopsies (Supplementary Fig. 3b), confirming earlier studies using sALS autopsy muscle^57^. This notion may also explain why the P56S-VAPB tg mice published so far (also included in this study), which express P56S-VAPB exclusively in the CNS, show only minor degeneration of the neuromuscular axis.

## Supplementary information

Supplementary Table 1

Supplementary Figure legends

Supplementary figure 1

Supplementary figure 2

Supplementary figure 3

## Data Availability

Available promptly upon request.
