# Supplementary material for: Pathomechanisms of ALS8: altered autophagy and defective RNA binding protein (RBP) homeostasis due to the VAPB P56S mutation
Source: Cell Death Dis. 2021 May 10;12(5):466. doi: 10.1038/s41419-021-03710-y (PMC8110809; doi:10.1038/s41419-021-03710-y)
Supplement: Supplementary file 1 — Supplementary Table 1 [file 41419_2021_3710_MOESM1_ESM.doc]

**Supplementary Table 1:** List of primary and secondary antibodies used in this study
 (Previously used by us in the references below: 1-5)

| **Antibody** | **Commercial source** | **Species** | **Reference** | **Working Dilution** | | |
| --- | --- | --- | --- | --- | --- | --- |
|  |  |  |  | **IHC/IF** | | **WB** |
| **Primary antibody** | | | | | | |
| Anti-GFP | Roche | mouse | 11804460001 | **-** | 1:1000 | |
| Anti-GADD | Santa Cruz | Rabbit | SC-793 | 1:100 | 1:1000 | |
| Anti-GRP78 (Bip) | BD Biosciences | Mouse | 610978 | 1:100 | 1:1000 | |
| Anti-p62 | MBL | Rabbit | PM045 | 1:100 | 1:50,000 | |
| Anti-p62 | Sigma Aldrich | Rabbit | P0067 | 1:100 | 1:50,000 | |
| Anti-LC3 | Sigma Aldrich | Rabbit | L7543 | 1:100 | 1:50,000 | |
| Αnti α-Tubulin | Sigma Aldrich | Mouse | T5168 | **-** | 1:10,000 | |
| Anti-VAPB | Home made | Rabbit | **-** | 1:50 | 1:1000 | |
| Anti-EGFR | Cell signalling | rabbit | 2232L | **-** | 1:1000 | |
| Anti-ULK-1 | Sigma Aldrich | Rabbit | HPA063990 | 1:100 | 1:1000 | |
| Anti-HSP70 | Milipore | Mouse | MAB3516 | 1:100 | 1:1000 | |
| Anti-HA | Santa Cruz | Mouse | Sc-7392 | 1:50 | **-** | |
| Anti-SigR1 | Proteintech | Rabbit | 15168-1-AP | 1:100 | **-** | |
| Anti-SigR1 | Santa Cruz | Mouse | Sc-166392 | 1:50 | 1:1000 | |
| Anti-Ubiquitin (ub) | Dako | Rabbit | Z0458 | 1:100 | 1:1000 | |
| Anti-pEIF2α | Cell signaling | Rabbit | 9721S | **-** | 1:1000 | |
| Anti-pTDP43 | Cosmo Bio Co. LTD | Mouse | TIP-PTD-M01 | 1:5000 | **-** | |
| Anti-TDP43 | abnova | Mouse | H00023435-M01 | **-** | 1:1000 | |
| Anti-TDP43 | abcam | Mouse | Ab57105 | 1:500 | **-** | |
| Anti-Matrin3 | Bethyl | Rabbit | IHC-00081 | 1:200 | **-** | |
| Anti-Matrin3 | Bethyl | Rabbit | A300-591A | 1:1000 | 1:1000 | |
| Anti-Fus | Novus Biologicals | Rabbit | NB100-2599 | 1:100 | 1:50,000 | |
| Anti-TIAR1 | BD Biosciences | Mouse | 610352 | 1:100 | 1:1000 | |
| Anti-human G3BP | BD Transduction | Mouse | 611126 | 1:100 | **-** | |
| Anti-TAF-15 | Bethyl | Rabbit | ICH-00094 | 1:500 | 1:1000 | |
| Anti-EWSR1 | ATLAS | Rabbit | HPA051771 | 1:500 | 1:1000 | |
| Anti-caspase-3 | Cell signaling | Rabbit | 9665 | 1:100 | **-** | |
| Anti-HSP27 | Cell signalling | Mouse | 2402S | 1:100 | **-** | |
| Anti-STX17 | Sigma Aldrich | rabbit | HPA001204 | 1:100 | 1:1000 | |
| Anti-ATG5 | Novus | rabbit | NB110-53818 | 1:100 | 1:1000 | |
| **Secondary antibodies** |  |  |  |  |  | |
| Poly HRP-GAMs/Rb IgG | Immunologic VWR | Ms/Rb | VWRKDPVB500HRP | Read-to-use |  | |
| Poly HRP-Anti Goat IgG | Immunologic VWR | Goat | VWRKDPVG110HRP | Read-to-use |  | |
| Biotinylated goat anti-mouse | Vector Laboratories | Goat | BA-9200 | 1:500 |  | |
| Alexa Fluor 488 goat anti-mouse | Life Technologies | Goat | A11001 | 1:500 |  | |
| Alexa Fluor 555 goat anti-mouse | Life Technologies | Goat | A21424 | 1:500 |  | |
| Alexa Fluor 488 goat anti-rabbit | Life Technologies | Goat | A11008 | 1:500 |  | |
| Alexa Fluor 546 goat anti-rabbit | Life Technologies | Goat | A11010 | 1:500 |  | |
| Alexa Fluor 488 donkey anti-goat | Life Technologies | Donkey | A11055 | 1:500 |  | |
| Goat anti-rabbit IgG (H+L), HRP | Thermo Scientific | Goat | 31460 |  | 1:10000 | |
|  |  |  |  |  |  | |

**REFERENCES**

1. Dreser A, Vollrath JT, Sechi A, Johann S, Roos A, Yamoah A *et al* (2017) The ALS-linked E102Q mutation in Sigma receptor-1 leads to ER stress-mediated defects in protein homeostasis and dysregulation of RNA-binding proteins. Cell Death Differ 24:1655-71.

2. Filezac de L'Etang A, Maharjan N, Cordeiro Brana M, Ruegsegger C, Rehmann R, Goswami A *et al* (2015) Marinesco-Sjogren syndrome protein SIL1 regulates motor neuron subtype-selective ER stress in ALS. Nat Neurosci 18:227-38.

3. Goswami A, Jesse CM, Chandrasekar A, Bushuven E, Vollrath JT, Dreser A *et al* (2015) Accumulation of STIM1 is associated with the degenerative muscle fibre phenotype in ALS and other neurogenic atrophies. Neuropathol Appl Neurobiol 41:304-18.

4. Jesse CM, Bushuven E, Tripathi P, Chandrasekar A, Simon CM, Drepper C *et al* (2016) ALS-Associated Endoplasmic Reticulum Proteins in Denervated Skeletal Muscle: Implications for Motor Neuron Disease Pathology. Brain Pathol. 6:781-794

5. Vollrath JT, Sechi A, Dreser A, Katona I, Wiemuth D, Vervoorts J *et al* (2014) Loss of function of the ALS protein SigR1 leads to ER pathology associated with defective autophagy and lipid raft disturbances. Cell Death Dis 12:243.
