# Supplementary material for: Pathomechanisms of ALS8: altered autophagy and defective RNA binding protein (RBP) homeostasis due to the VAPB P56S mutation
Source: Cell Death Dis. 2021 May 10;12(5):466. doi: 10.1038/s41419-021-03710-y (PMC8110809; doi:10.1038/s41419-021-03710-y)
Supplement: Supplementary file 2 — Supplementary Figure legends [file 41419_2021_3710_MOESM2_ESM.docx]

## Supplementary Figure 1

**(a)** Abnormal VAPB aggregates (arrowheads) of P56S-VAPB and normal ER localization of wt-VAPB (left panel) after overexpression in NSC-34, HeLa cells and in RCMH (human muscle cell line) cells. Scale bar = 15 µm.

**(b)** Human IPSc derived MNs transfected with GFP- wt-VAPB and P56S-VAPB, showing aggregates in P56S-VAPB transfected MNs (right panel).

**(c)** Immunofluorescence staining using HA antibody in lumbar spinal cord α-MNs of wt and P56S-VAPB tg mice, showing globular aggregates in the α-MNs (arrowheads) of P56S-VAPB tg mice. Scale bars: 15 µm.

**(d)** Routine stained sections from the muscle biopsy of the patient´s mother, who was also affected by muscle wasting, showed grouped neurogenic muscle fibre atrophy (arrows). Cryostat sections; scale bars = 50 µm.

**(e)** DAB immunohistochemistry performed on ALS8 muscle biopsy showing increased accumulations of ER chaperones (SigR1, GRP78, and HSP27), proteostasis marker (HSP70 and ubiquitin) in atrophic and partially atrophic fibres. Note the large autophagic vacuoles (white arrows) present in hypertrophic fibres. Scale bars: 60 µm.

**(f)** Control and ALS8 patients’ fibroblasts were treated either with ER stress Thapsigargin (2 µM) or proteasome inhibitor MG132 (2 µM) for 4 h and then processed for immunoblot analysis. Note the increased gel top aggregation of mutant VAPB in ALS8 fibroblasts together with additionally increased ER stress after the treatment of known ER stressor Thapsigargin.

**(g)** Immunoblot analysis of RCMH cells over-expressing wt-VAPB and P56S-VAPB. Note the increased levels of ER / cytosolic chaperones and of LC3II along with increased levels of ubiquitin conjugate in cells transfected with P56S-VAPB.

**(h)** Co-labelling of wt-VAPB and P56S-VAPB with p62 in NSC-34 cells, showing globular accumulations of p62 and its co-localization with P56S aggregates. Scale bar = 10 µm.

**(i)** Human IPSc derived MNs transfected with GFP- wt-VAPB and P56S-VAPB, showing globular accumulation of LC3 Puncta (arrow heads; lower right panel) co-localizes with P56S aggregates. Scale bar = 10 µm.

**(j)** Immunoblot analysis showing concentration dependent increased levels of LC3II and p62 in P56S-VAPB overexpressing cells compared to the wt-VAPB expressing NSC-34 cells.

**(k)** Human IPSc derived MNs transfected with GFP- wt-VAPB and P56S-VAPB, showing increased neuronal death in P56S-VAPB overexpressing neurons. Scale bar = 10 µm.

## Supplementary Figure 2

**(a)** a-d: Axonal spheroids (white arrows) in P56S-VAPB tg mice spinal cord, filled with accumulated cell organelles (mostly mitochondria and vacuoles of various size) and autophagic vacuoles. Others (b-c) show prominent tubular proliferation (black arrow) of the axoplasmic reticulum. (e-f) Neuronal cell bodies also show osmophilic tubular aggregations (black arrows), mostly connected to the endoplasmic reticulum. Scale bars: 1.5 µm)

**(b)** Double immunofluorescence labelling for HA and for the ER chaperones SigR1, GRP78 and Ubiquitin in lumbar spinal cord α-MNs of P56S-VAPB tg mice, showing co-localization (arrowheads) with P56S VAPB aggregates. Scale bars: 15 µm.

**(c)** Representative DAB immunohistochemistry (Left panel) and immunofluorescence staining (right panel) using HA antibody in wt-VAPB and P56S-VAPB tg mice brain, showing globular aggregates in cortex and hippocampus of P56S – VAPB tg mice brain. Scale bars: 15 µm.

**(d-f)** Double immunofluorescence labelling for HA and for the ER chaperone SigR1 **(d)** autophagy marker p62 **(e)** and for ER stress induced transcription factor GADD-153/CHOP **(f).** Note the increased levels of SigR1, p62 and GADD-153 and co localization of SigR1 and p62 with globular P56S-VAPB aggregates (arrowheads) in cortical neurons of P56S-VAPB tg mice. Scale bars: 15 µm.

**(g)** DAB immunohistochemistry using antibodies against HA, VAPB and HSP70, showing increased immunoreactivity of the degenerating cortical neurons. Scale bars: 15 µm.

**(h)** Immunoblot analysis of total brain homogenates from 200 days old P56S-VAPB (n=9,male 200days) and their age matched wt-VAPB mice (n=3,male 201 days) showing an overall increased level of autophagy markers p62 and LC3 together with increased ubiquitin conjugates in P56S-VAPB tg mice brain. Note the gel-top aggregation of p62 and ubiquitin (red arrowheads) in majority of P56S-VAPB tg mice brain. Unpaired Student’s t-test for comparison between two sample groups (average of n=3 wt-VAPB mice and average of n=9 P56S-VAPB mice) Values were expressed as mean ± standard error of mean (SEM) from three independent blots. The asterisks (*) denote significant differences (**p*<0.05).

## Supplementary Figure 3

**(a-b)** Immunofluorescence analysis of HeLa cells after treatment with Bafilomycin A (200nM) for 4 h. Massive accumulation of LC3 and p62 **(a)** Cytoplasmic mis-localization of FALS associated RBPs (FUS, TDP-43, Matrin 3, TAF-15 and EWRS1 **(b)**. Scale bar = 10 µm.

**(c)** Immunoblot analysis of the subcellular fractions obtained from HeLa cell after treatment with Bafilomycin A (200nM) for 4 h. Note the increased levels of RBPs (FUS, TDP-43, Matrin 3, TAF-15, and EWSR1) in the cytoplasmic extract (CE; red arrowheads) of Baf. A treated sample. ME; membrane extract, NE; nuclear extract, CB; chromatin bound and PE; cytoskeleton respectively.

**(d)** Representative DAB immunohistochemistry showing granular pTDP-43 immunoreactivity (arrows) in atrophic, denervated muscle fibres in the sALS muscle biopsy compared to the mild myonuclear immunolabelling in normal sized fibres in control. (n=4 controls, n= 4 sALS, biopsies). Scale bars = 50 µm.

**(e)** Group of atrophic muscle fibres in an ALS8 case showing disintegration of myofibrils. Focal accumulations of glycogen granules (black arrows), lipofuscin granules, autophagic vacuoles and other organelles (white arrows) in the ALS8 muscle biopsy. Scale bar = 4 µm. Lower panel; ER widening (arrows) associated with altered nuclear envelope structure (arrowhead). Electron microscopy; scale bar = 300 nm.
