# Supplementary figures and images for: Pathomechanisms of ALS8: altered autophagy and defective RNA binding protein (RBP) homeostasis due to the VAPB P56S mutation
Source: Cell Death Dis. 2021 May 10;12(5):466. doi: 10.1038/s41419-021-03710-y (PMC8110809; doi:10.1038/s41419-021-03710-y)

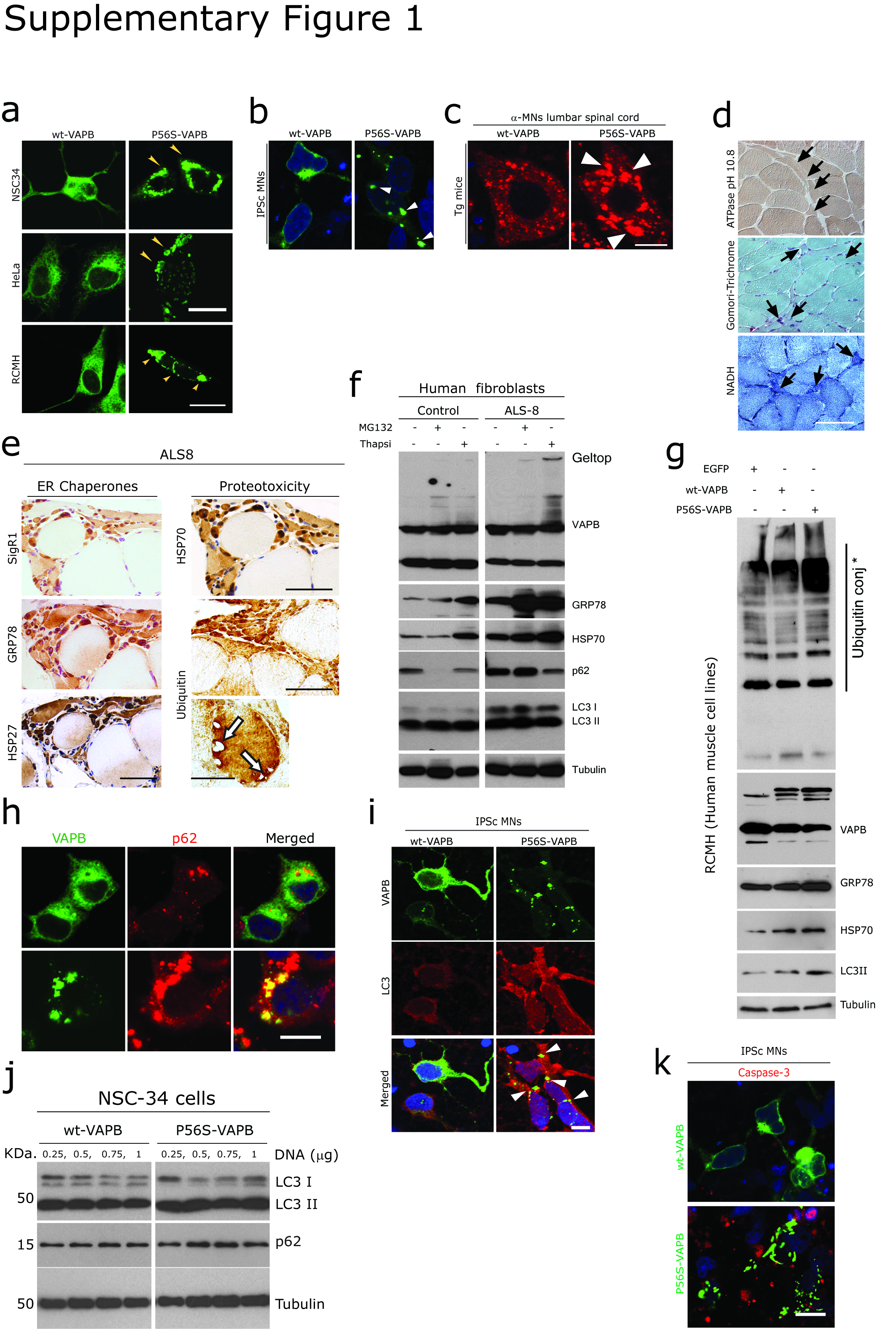

Supplement: Supplementary file 3 — Supplementary figure 1 [file 41419_2021_3710_MOESM3_ESM.tif]

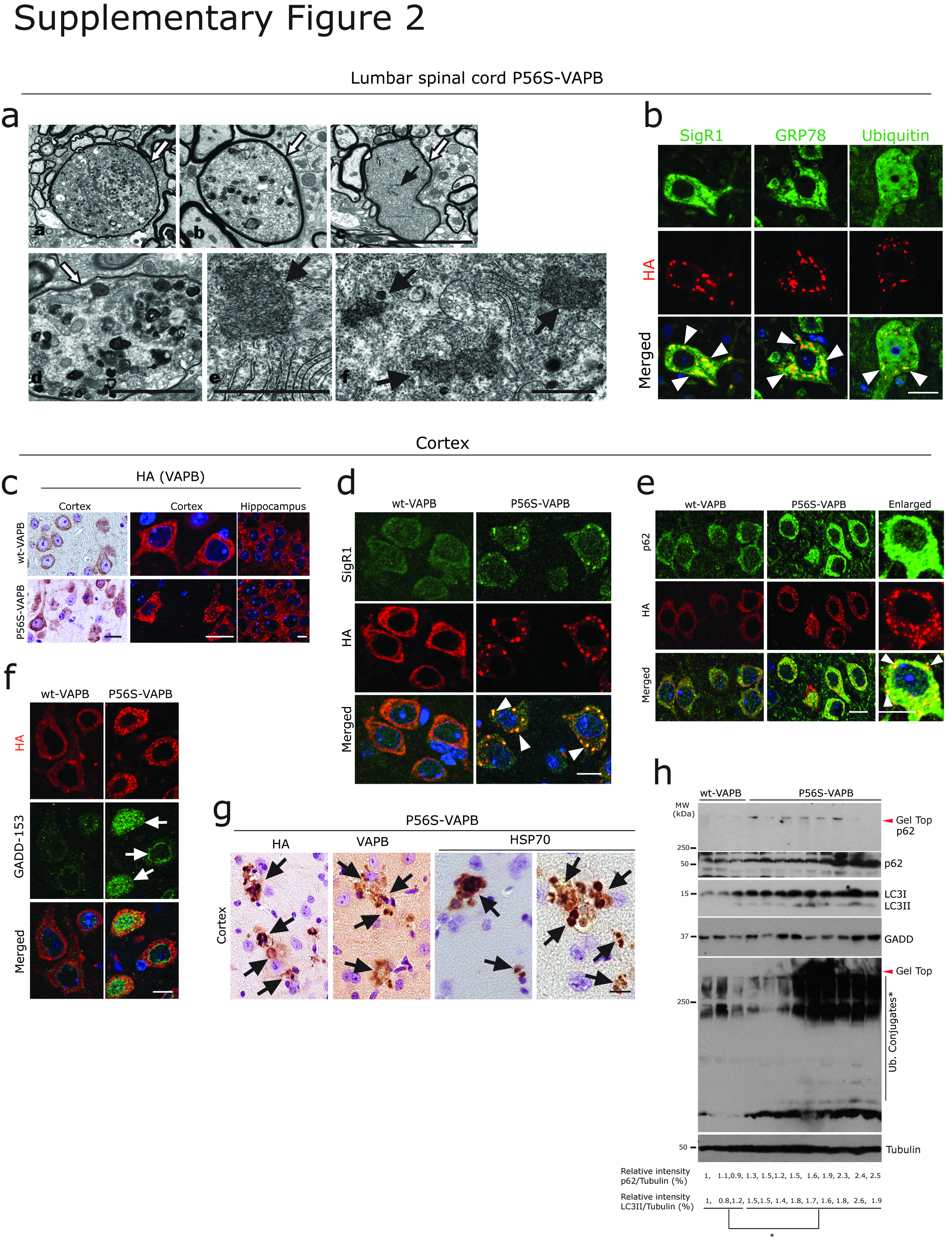

Supplement: Supplementary file 4 — Supplementary figure 2 [file 41419_2021_3710_MOESM4_ESM.tif]

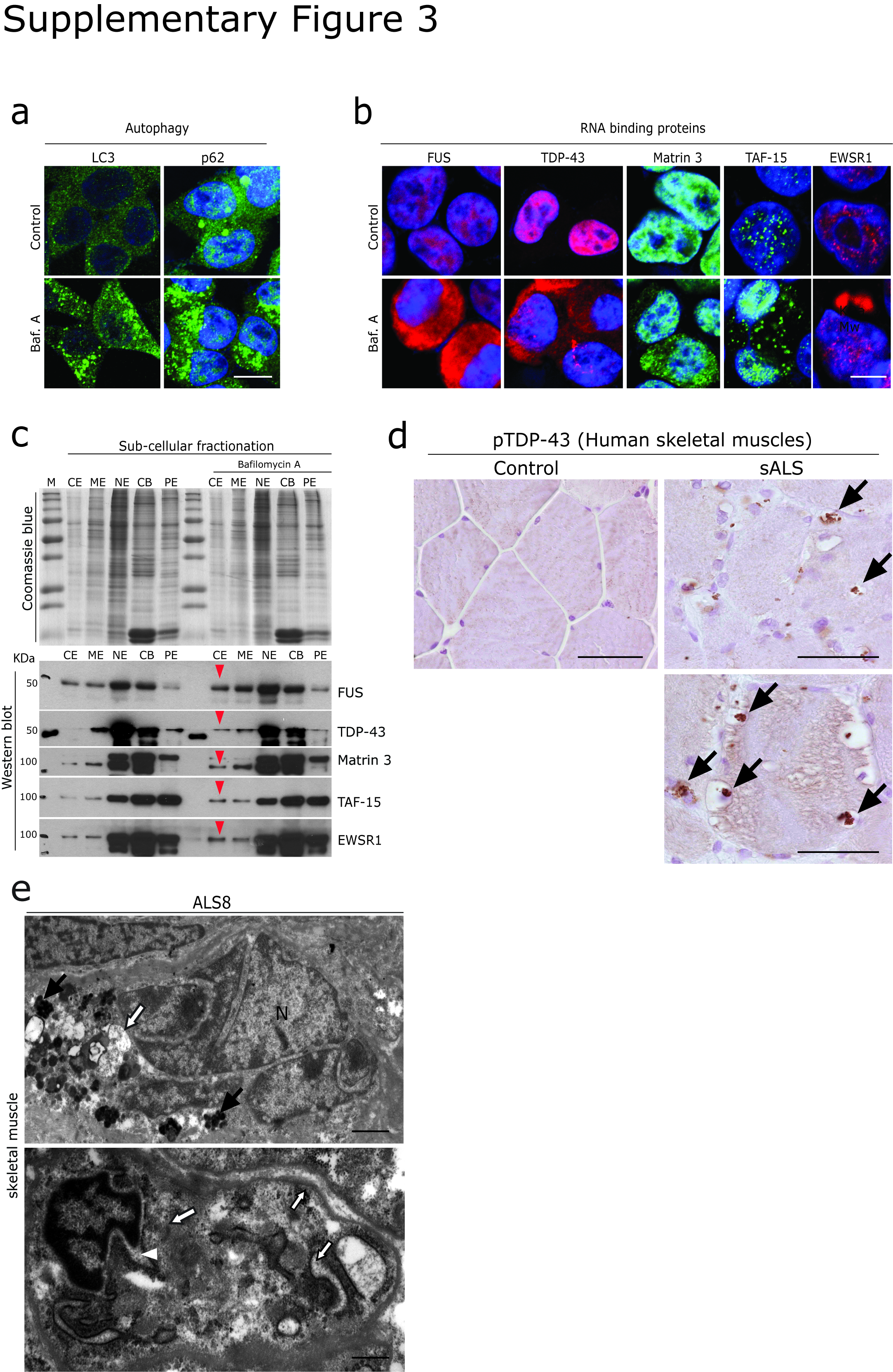

Supplement: Supplementary file 5 — Supplementary figure 3 [file 41419_2021_3710_MOESM5_ESM.tif]
